# Surgical strategies in elderly patients with acute type A aortic dissection—a systematic review

**DOI:** 10.3389/fsurg.2026.1856125

**Published:** 2026-07-24

**Authors:** Vanessa I. T. Zwaans, Lina Hülsenberg, Leonhard Wert, Jasper Iske, Gaik Nersesian, Julius Kaemmel, Martina Dini, Matteo Montagner, Markus Kofler, Semih Buz, Volkmar Falk, Jörg Kempfert, Leonard Pitts

**Affiliations:** 1 Department of Cardiothoracic and Vascular Surgery, Deutsches Herzzentrum der Charité (DHZC), Berlin, Germany; 2 Department of Cardiothoracic and Vascular Surgery, Charité—Universitätsmedizin Berlin, Corporate Member of Freie Universität Berlin and Humboldt-Universität zu Berlin, Berlin, Germany; 3 DZHK (German Centre for Cardiovascular Research), Berlin, Germany

**Keywords:** aorta, elderly, endovascular, malperfusion, stent implantation, surgery, type a aortic dissection (TAAD)

## Abstract

Acute type A aortic dissection (ATAAD) is a life-threatening emergency requiring urgent surgery. Advanced age is a major risk factor for operative mortality and strongly influences treatment decisions. This systematic review evaluates perioperative characteristics, surgical strategies in regard to outcomes and mortality in patients older than 70 years undergoing ATAAD repair. A PRISMA-guided database search identified original studies published between 2000 and 2025. Early and late outcomes were pooled, with thirty-day mortality as the primary endpoint and postoperative complications, organ dysfunction and quality of life as secondary endpoints. Key aspects of ATAAD management, including patient selection, extent of repair, cerebral protection and perioperative organ protection strategies, were systematically analyzed. Of 1,910 screened studies, 41 publications including 3,969 elderly patients were analyzed, encompassing randomized, observational and case-control designs. Preoperative malperfusion occurred in 22.8% of patients, most commonly cerebral (16.7%) and renal (13.5%). When analyzed within their respective cohorts, the majority of patients received standard of care ascending aorta or hemiarch replacement. This was followed by total arch replacement and root replacement. Mean cardiopulmonary bypass, cross-clamp and circulatory arrest times were 201, 104 and 45 min, respectively, at 23 °C. Postoperative morbidity was substantial, with neurological complications (12%), delirium (30%), respiratory failure (21%) and dialysis-dependent renal failure (22%). 30-day mortality was in average 21% and was mainly due to bleeding, low cardiac output and multiple organ failure. Long-term quality of life, assessed by SF-36, remained satisfactory. However, data was overall heterogeneously presented and therefor limited interpretability concerning the addressed endpoints. ATAAD surgery in elderly patients remains challenging due to frailty and comorbidities. Careful patient selection and treatment in specialized centers are essential. Despite the emerging hybrid and endovascular approaches, open repair was the most commonly reported intervention. The available data suggests that advanced age was not consistently associated with withholding surgical treatment, especially in cases of absence of malperfusion

## Introduction

Acute type A aortic dissection (ATAAD) represents a life-threatening diagnosis that carries a high risk of mortality (1). Advanced age is a well-known independent risk factor for increased mortality in ATAAD. According to European registry data (GERAADA), septuagenarians (70–79 years of age) show a perioperative mortality of around 16%, whereas octogenarians (80–89 years of age) show substantially higher early in-hospital mortality of about 30% (2). In addition to age, other factors such as center volume and surgical expertise, presence of malperfusion, the extent of repair and patient morbidity are major modifiers (3).

Elderly patients have several additional vulnerabilities compared to younger patients such as impaired renal function, higher incidence of malperfusion, frailty, poor tissue quality and further comorbidities which contribute to their increased susceptibility in case of prolonged cardiopulmonary bypass (CPB), hypothermic circulatory arrest (HCA) and overall long operative times (4).

With the general increase in human life expectancy and improved healthcare, an increasing number of elderly patients are being referred for aortic surgery. Defining clear clinical outcomes in this cohort may provide further insight into potential risk factors and outcome patterns relative to younger patients. These findings may inform ongoing discussions about the use of open surgery in elderly patients under varying clinical circumstances (1).

Simultaneously, patient-reported outcome measures (PROMs) are gaining scientific interest, particularly since operative mortality has not decreased significantly over the past years in surgery of ATAAD. Factors such as quality of life (QoL), physical independence, emotional well-being and social integrity represent indispensable components for understanding the overall effectiveness and long-term impact of surgical management in this population (5).

The optimal treatment strategy for ATAAD in elderly patients remains a matter of debate, reflecting the complexity of decision making regarding the extent of aortic repair. This mainly depends on the surgeon's experience and intraoperative judgement, but may have a substantial impact on the outcome in elderly patients (6).

Patient selection and the choice of surgical approach may predict postoperative outcomes. The extent of preoperative malperfusion is a vital factor in planning perioperative organ protection and anticipating potential postoperative organ dysfunction (7). Where elderly patients present multiple malperfusion syndromes, mortality and morbidity rise tremendously (8).

This systematic review aims to summarize and describe the clinical outcomes of patients aged 70 years and older undergoing surgery for ATAAD in current literature to evaluate and discuss important factors contributing to an increased surgical risk.

## Methods

The literature research, concept, inclusion criteria, research question and hypothesis of this review were defined according to the Preferred Reporting Items for Systematic Reviews and Meta-Analyses (PRISMA) guidelines (Supplementary Figure 2) (9). This systematic review was conducted in accordance with established methodological principles for systematic reviews as outlined in the Cochrane Handbook for Systematic Reviews of Interventions (10). However, a formal meta-analysis was not performed due to substantial heterogeneity in study design, patient characteristics and outcome definitions in the included studies. Furthermore, there was a lack of consistently extractable outcome data suitable for effect size calculation.

### Search strategies

Applying the according mesh terms, systematic research was carried out on PubMed, OVID/Medline and Web of Science until 30 November 2025. Mesh terms applied in the given databases can be found in the Supplementary Materials (Figure 2). The results of this research were screened and included, provided they met the inclusion criteria of operative outcome in acute type A aortic dissection in patients ≥ 70 years at time of the operation. Matching publications were then imported into the reference management software EndNote®. Two independent authors reviewed all full texts of the included publications (Supplementary Table 1).

### Inclusion criteria

Published case series as well as retrospective, observational or randomized clinical trials (RCT) among patients ≥ 70 years at time of the operation diagnosed with type A aortic dissection as the main indication for surgical intervention were considered. This review included publications from the last 23 years (2001–2025).

Literature not written in English or German, poorly described case reports, review articles and studies not providing relevant data were excluded. Studies were excluded from the analysis if data were in a non-extractable format, duplicated or if the research was conducted in an animal model. Studies were excluded, for example, if patients were all younger than 70 years or populations included mixed-age cohorts where patients older than 70 years could not be extracted, if studies reported on type B aortic dissection only or on chronic aortic dissection rather than acute dissection (Supplementary Figure 2). Two assessors (LP and LW) independently reviewed the titles and abstracts of potentially eligible studies and selected studies that met the inclusion and exclusion criteria for full-text retrieval and further examination. Any disagreements between the two reviewers regarding study eligibility were resolved through discussion and, if unresolved, consultation of a third reviewer (JI) to make the final decision. This approach was conducted in accordance with PRISMA guidelines. Data extraction

The extracted data were collected by the first author and reviewed for accuracy by the last author. Data extracted included authors, title, year of publication and patient demographics (age, sex). Further data were subdivided into subgroups: preoperative, perioperative and postoperative patient data, causes of in-hospital death, discharge location and postoperative QoL category, all of which were extracted and recorded in Microsoft Excel 2024 (Microsoft Corp., Redmond, WA, USA). In order to receive pooled analyses, study-level averages of the included studies were aggregated into one dataset. Descriptive summary measures were then calculated from the data. There was no application of sample-size weighting or meta-analytic models, therefore all studies contributed equally to the pooled estimated. Given the heterogeneity of the included studies and the inconsistent reporting of variables, a formal meta-analysis was deemed inappropriate. The extracted study-level data was therefor synthesized descriptively while respecting the original data reported in the included studies.

Preoperative patient characteristics included BMI, EuroSCORE II, classification of DeBakey Type I or II, intramural hematoma (IMH), presence of bicuspid aortic valve (BAV), Marfan syndrome, aortic rupture, pericardial tamponade, preoperative shock and the need for preoperative resuscitation as well as preoperative malperfusion categorized as cerebral (C)/neurological (N), myocardial (M), visceral (V), iliofemoral (I), mesenteric (SMA/celiac) or renal. Comorbidities such as coronary artery disease (CAD), peripheral arterial occlusive disease (PAD), arterial hypertension (AH), diabetes mellitus, chronic obstructive pulmonary disease (COPD), renal failure requiring hemodialysis, history of smoking and/or alcohol consumption, occurrence of aortic valve regurgitation that was more than moderate, aortic valve stenosis, previous cardiac surgery, preoperative atrial fibrillation (AF), congestive heart disease, previous myocardial infarction, history of stroke, preoperative creatinine level and preoperative ejection fraction (EF) were also included.

Perioperative patient data consisted of information relating to perfusion such as circulatory arrest time, mean core temperature, isolated cerebral perfusion time and cerebral perfusion flow. Information about the extent of the surgical intervention included total aortic arch repair, replacement of the ascending aorta and hemiarch, total arch replacement, (Frozen) Elephant Trunk procedure, aortic root reconstruction, replacement via reconstruction using a Bentall/aortic valve conduit, or aortic root untouched, aortic valve replacement (AVR) and need for coronary artery bypass graft (CABG). Operative times, cardiopulmonary bypass times (CPB), femoral, axillary or central aortic cannulation strategies were extracted.

Postoperative data consisted of the occurrence of adverse events (AE), including infection, sepsis, delirium, cerebral, due to bleeding, new-onset atrial fibrillation, respiratory failure, renal failure requiring hemodialysis, tracheotomy, transient or permanent neurologic deficit, gastrointestinal bleeding (GIB), occurrence of limb ischemia, postoperative myocardial infarction and the need for readmission. Intensive care unit (ICU) stay was documented alongside ventilation time, need for reintubation, prolonged ventilation (>48 h), chest tube drainage (mL/24 h), need for re-exploration due to bleeding and length of total stay (LOS).

Causes of in-hospital death were manifold and included, among others, multiple organ failure, respiratory failure, sepsis, intracranial hemorrhage, GIB, aortic rupture, tamponade, coronary malperfusion, hypovolemic shock and bleeding in general.

Discharge locations were inconsistently documented. If at all, a distinction was made between transitional care unit or rehabilitation, home, hospice, nursing home, another hospital or missing data.

Three studies examined patients' quality of life after surgery, one without specification of the assessment instrument (11) and two employing the SF-36 questionnaire (12, 13). QoL is usually assessed through validated patient-reported outcome measures (PROMs) such as the most commonly used Short-Form-36 (SF-36) and -12 (SF-12), EQ-5D and PROMIS (Patient-Reported Outcomes Measurement Information System) (5, 14). QoL was categorized as physical functioning, role limitations (physical), energy/fatigue, emotional wellbeing, social functioning, pain and general health.

In general, the reviewed studies showed considerable clinical and methodological heterogeneity including study design, patient populations, operative techniques and study periods. Due to the lack of specific focus amongst the included studies as well as inconsistent reporting of relevant variables, it was not attainable to create predefined subgroup, stratified and sensitivity analysis. Therefore, data were interpreted accordingly to the present heterogeneity of variables.

### Outcomes

The aim of this systematic review was to summarize patient characteristics and analyze surgical outcomes of treating aortic type A dissection in the elderly aged older than 70 years. It included aspects such as cerebral protection strategies and neurological outcome as well as perioperative organ protection. Based on these findings, the impact of comorbidities, anatomic constitutions, surgical strategies and alternative treatment concepts that might influence postoperative outcomes, survival and QoL was discussed.

## Results

### Literature research

Of the articles retrieved for evaluation, 1,910 (including 904 duplicates) met the inclusion criteria based on the abstract; of these, 1,006 were identified as relevant and full-text reading was performed. Ultimately, 917 articles were excluded because they did not meet the inclusion criteria or were not written in English or German or were case reports. In addition to the 89 articles extracted from PubMed, Ovid and Web of Science, 17 articles were included as secondary literature items. See the list of publications (Supplementary Table 1).

Study populations varied in quantity and not all publications comprehensively provided preoperative, perioperative and postoperative data. Therefore, not every patient group was screened for all factors associated with possible adverse events in TAAD. Operative variables were not reported in a standardized manner across the reviewed studies. Therefor, percentages for given surgical strategies were calculated using the number of patients for whom data on the respective variable were available. This is why denominators vary across reported operative strategies.

After full-text evaluation, 41 articles providing individual data pertaining to 7,481 cases were included. The publications spanned a period of 24 years (Supplementary, Figure 3).

The included studies were retrospective cohort studies, multicenter studies or comparative studies focusing mainly on surgical treatment and outcomes of ATAAD in the elderly. Most publications focused on postoperative outcomes in general, whereas one study focused on neurological outcomes after ATAAD surgery (15) and two studies investigated the impact of preoperative malperfusion (8, 16).

A dataset (Supplementary Table 2a–h) of 7,481 patients of mixed origin was ultimately obtained; of those assessed, 1,493 (53%) were female and 1,347 (47%) were male.

The data collection process differed, and not all details were provided by all publications (Supplementary, Table 2a-h, 3). Data comparing 30-day mortality was collected from 2,840 patients (Supplementary, Table 3).

### Baseline patient characteristics

The average age was 77.92 years. The mean BMI for the patient data provided was 25.18 kg/m^2^ (±2.59) and the mean EuroSCORE II was 20.3 ± 15.6.

Concerning the classification of aortic aneurysms, where assessed, 59.4% had DeBakey type I and 42.6% had DeBakey type II dissection. Intramural hematoma (IMH) was seen in 38.7% of patients, 3.1% of patients had bicuspid aortic valves and 0.6% had Marfan syndrome. In total, of all patients diagnosed with acute type A aortic dissection, 32.9% were in preoperative shock and 32.9% showed pericardial tamponade at the time of arrival in hospital. 30.7% of aortic dissections showed signs of aortic rupture. 5.8% of patients needed preoperative resuscitation.

Comorbidities included peripheral artery disease (PAD) which was present in 23.6% and history of stroke was documented in 11.5% of patients. The most common comorbidity was arterial hypertension with 75.8%. Diabetes mellitus was present in 11.8% and chronic obstructive pulmonary disease in 12.0% of patients. Concerning substance use, 20.1% showed prior or ongoing smoking and 9.5% showed regular alcohol consumption. Pre-existing renal failure requiring hemodialysis was reported in 11.8% of patients.

Cardiac comorbidities such as diagnosed coronary artery disease (CAD) in 20.0% of patients and a history of myocardial infarction in 9.8%. Congestive heart failure was seen in 60.4%, and 15.6% of patients showed preoperative atrial fibrillation.

The most assessed laboratory value was creatinine with a mean of 1.33 ± 0.36 mg/dL. Preoperative left ventricular ejection fraction (LVEF) was 56.0 ± 7.4.

A total of 31.0% of patients showed preoperative malperfusion. Some studies did not provide a description of the type of malperfusion. Those which did reported 16.7% cerebral malperfusion, 5.7% coronary malperfusion, 6.1% visceral malperfusion, 13.5% renal malperfusion and 8.1% limb malperfusion.

### Perioperative data

In the respective cohorts, the standard care was replacement of the ascending aorta or hemiarch in 48.7% of patients (1,048/2,152), followed by total arch replacement was performed in 15.5% of patients (483/3,113) and root replacement in 11.4% of patients (129/1,129). Aortic root replacement was performed in 10.2% of patients (328/3,224), whereas conservative aortic root reconstruction or repair was observed in 50.4% (903/1,791). Root replacement was most represented by the Bentall procedure, with an aortic valve conduit applied in 11.4% of patients (129/1,129). In comparison, isolated aortic valve replacement was required in 26.1% (446/1,711). Repair of both the ascending and descending aorta using the elephant trunk technique was reported in one publication, in which 95% of the patients underwent frozen elephant trunk (FET) repair (17). In addition to aortic surgery, coronary artery bypass grafting due to coronary malperfusion was performed in 9.5% of patients (212/2,234). Since reporting of operative strategies varied across reviewed studies, these proportions were derived from variable denominators and should not be interpreted as representing a single unified patient cohort.

The mean operating time was 347.2 ± 163.0 min, of which 200.6 ± 57.78 min were cardiopulmonary bypass time, 45.4 ± 41.6 min in circulatory arrest, 103.6 ± 28.9 min cross-clamping and 60.3 ± 75.2 min isolated cerebral perfusion time. During circulatory arrest the mean core temperature was 23.1 ± 3.2 °C. Cannulation for cardiopulmonary bypass was either central in 14 out of 406 patients, or more commonly via femoral vessels in 457 out of 1,005 patients or via axillary vessels in 234 out of 777 patients.

The mean stay on the intensive care unit (ICU) was 10.9 ± 16.4 days. Here, the average ventilation time was 67.7 ± 33.8 h, 6.0 ± 3.0 patients had to be reintubated and 39.0 ± 42.0 had prolonged ventilation (>24 h). Mean chest tube drainage was 817.88 ± 364.75 mL in 24 h. Due to bleeding 8.0 ± 15.0 patients underwent re-exploration after the primary surgery. The total length of stay in hospital was 23.9 ± 12.7 days.

### Postoperative data

Postoperative complications were most commonly neurological, with 30.0% of patients developing postoperative delirium and 12.0% suffering cerebral complications, of which 7.0% were transient neurological deficits and 4.0% permanent deficits such as hemiplegia or visual field defects.

After surgery, 12.0% of patients suffered cerebral stroke, 12.0% of patients showed infectious complications with 10.0% in septic shock. Gastrointestinal bleeding due to malperfusion was seen in 3.0% of patients and 1.0% of patients showed limb ischemia. Bleeding, which was not further defined, was seen in 16.0% of patients. Renal failure necessitating hemodialysis was seen in 22.0% of patients and 21.0% suffered from respiratory failure leading to tracheotomy in 10.0% of patients. New-onset atrial fibrillation was reported in 25.0% of patients and postoperative myocardial infarction was seen in 3.0%. After discharge, 10.0% of patients had to be readmitted to hospital.

Causes of postoperative in-hospital death were documented irregularly. In 13% low cardiac output, in 5.0% of patients multiple organ or respiratory failure led to death, whereas aortic rupture was the cause of death in 4.0% of patients. Septic, cardiac or hypovolemic shock or uncontrollable postoperative bleeding was the cause of death in 3% of patients. Intracranial hemorrhage and cardiac tamponade in 1.0% of patients. The pooled 30-day-mortality was in average 21.0%. Individual study estimates ranged from 8% to 40%, reflecting substantial heterogeneity among study populations and treatment strategies.

The assessment of QoL after surgery is an important tool to evaluate not only survival but also functional recovery as well as physical and mental well-being. However, the assessment of QoL was scarce with only three publications addressing this aspect – one without specifying the assessment instrument (11) and two employing the SF-36 questionnaire (12, 13). In SF-36, the assessment consists of 8 scales and scores on each domain range from 0 (worst possible health status) to 100 (best possible health status). The average score for physical functioning was 58.3 ± 8.0, for role limitations due to physical health 69.0 ± 31.2, for vitality meaning energy or fatigue level 50.3 ± 2.5, for emotional well-being 71.9 ± 12.9, for bodily pain 60.9 ± 22.6, and general health scored on average 54.6 ± 2.3.

## Discussion

This systematic review included 41 studies with a total of patients aged 70 or older undergoing surgical repair for acute type A aortic dissection. Overall, the available data suggests that surgical treatment can be performed with acceptable outcomes. However postoperative mortality remains present. Concerning operative approaches and outcome, data remains mostly heterogenous across the reviewed studies.

### Patient selection in ATAAD

Effective treatment of ATAAD in elderly patients requires thoughtful selection of individuals who are best suited for a specific surgical approach. A patient's age cannot be the only factor in decision making, and it has been demonstrated that satisfactory results can be achieved in septua- and octogenarians in the absence of severe malperfusion and pronounced comorbidities (11, 18). Physiological activity and frailty are stronger predictors for surgical outcome and mortality. The EuroSCORE II and the STS score may support identifying patients at risk. However, these scoring systems were not specifically developed for ATAAD and may not accurately reflect a patient's course (19). If scoring systems are used, it is strongly recommended to assess the surgical risk with the GERAADA score which is currently the only available and validated risk score for 30-day mortality in the surgical treatment of ATAAD. Other scoring systems are based on patient cohorts with different cardiac diseases, which leads to misinterpreted 30-day mortality predictions in patients with acute type A dissection (19). Besides this, comorbidities play a pivotal role in assessing a patient's operative risk. High-risk diseases such as severe COPD, advanced chronic kidney disease and history of stroke as well as severe cognitive impairment may show a tendency to delayed recovery after surgery (20).

The extent of dissection with signs of malperfusion syndromes as well as pericardial tamponade or aortic rupture may further enhance the perioperative risk. These patients might benefit more from interventional repair rather than surgery (21). ATAAD patients requiring preoperative cardiopulmonary resuscitation (CPR) have a very high mortality, but survival depends strongly on the cause of the cardiac arrest. Patients with tamponade at arrival show better long-term outcomes likely because tamponade can be rapidly and effectively reversed with pericardial drainage and urgent surgery (22).

Despite surgery being the treatment of choice in ATAAD, the evaluation of interventional options may be justified to avoid open central repair in elderly patients with several comorbidities and a consequently high perioperative risk (23). Any endovascular approach must be individualized since patient anatomy, particularly landing zones or kinking, varies substantially. Therefore, stent grafts must be highly customized, and their implantation requires center-specific expertise (24). Besides this, optimal medical therapy represents another alternative to surgery (25). Nevertheless, it remains challenging to distinguish those who may benefit from surgical intervention from those in whom no available strategy —despite timing, technique, or institutional expertise— can improve the outcome. Although palliative management does not play a substantial role in cardiac surgical therapy, it will increasingly gain relevance as we treat more patients of advanced age (26). Therefore, careful clinical assessment and an understanding of the patient's condition remain key aspects in treating ATAAD.

### Stroke and delirium

Postoperative stroke is strongly associated with worse outcomes such as higher rates of pneumonia, respiratory failure, or longer hospital stay, and those with a history of stroke are more likely to experience postoperative stroke (27, 28). A large-scale analysis of the STS database identified cannulation method as a key modifiable risk factor, with femoral cannulation showing an increased risk of stroke, while axillary cannulation was associated with a reduced risk (29). This review was unable to outline an association between postoperative stroke and mortality due to the multifactorial nature of postoperative death. However, mortality rates in stroke patients have been shown to be higher than in non-stroke patients. This emphasizes that permanent neurologic deficits lead to worse late survival and represent an immense impact on QoL (30). Another important aspect is postoperative delirium (POD) which is frequently observed in elderly patients undergoing ATAAD surgery, even in the presence of prolonged selective cerebral perfusion and optimized preoperative management (31, 32). Prolonged CPB and long operating times are well established contributors to POD, as is an extended ICU stay (33, 34). Shi et al. identified antegrade selective cerebral perfusion time as an independent risk factor, suggesting that prolonged or qualitatively impaired cerebral perfusion may predispose patients to delirium. Additional aspects such as cerebral oxygen delivery, microembolic risk, and preoperative neuropsychiatric vulnerability remain clinically plausible contributors (33).

Andrasi et al. emphasize that intraoperative variables such as hypothermia, hemodilution, blood gas imbalances and perfusion pressure likely influence neurologic outcomes. However, the available evidence is heterogenous (31). Thus, despite biologically plausible mechanisms such as ischemia-reperfusion injury and temperature-related metabolic stress, the literature to date does not permit final conclusions about whether the degree of hypothermia or specific cerebral protection strategies significantly modify the POD risk in the setting of ATAAD (32, 35).

### Detection of malperfusion via CTA

Accurate preoperative assessment of malperfusion via computed tomography in ATAAD patients is essential for adequate surgical decision-making (36). With high sensitivity (100%) and high specificity (98%), ECG-gated CTA represents the fastest and most appropriate tool for diagnosing ATAAD (37). This assessment goes beyond standard axial CT images (38). Aortic dissection is a three-dimensional process; therefore, relying solely on axial views bears the risk of overlooking key information such as the true and false lumen, entry tears, branch-vessel involvement and subtle intimal flaps (39). Multiplanar reconstructions (MPR) and centerline-based curved planar reconstructions should always be performed (40). Multidetector CTA provides high-resolution images and reduces artifacts due to its faster volume acquisition. Visualization should extend from the supraaortic vessels, ideally including the circle of Willis, to the femoral arteries to provide a comprehensive overview of the extent and affected aortic side branches (37). Besides this, multiplanar reconstruction of 3D datasets is indispensable for a full assessment of the dissection including measurement of the true and false lumen diameter as well as location of entries, re-entries and signs of endorgan malperfusion (see Supplementary Figure 4) (40).

Centreline analysis represents a semiautomated tool that enables accurate measurements perpendicular to the aortic axis after placing manual seed points (40). It supports decision-making on the extent of repair and aids planning of endovascular and hybrid procedures (39, 40).

Regardless of individual or institutional variations, ECG-gated CTA surveillance is recommended at 6 and 12 months, and subsequently annually for up to 5 years if the condition is stable thereafter (21).

### The prognostic significance of malperfusion

End-organ ischemia as a result of true lumen obstruction contributes to increased operative mortality. Prognoses are worst when malperfusion affects the mesenteric arteries (SMA and celiac trunk) leading to gut ischemia (41, 42). The STS database shows that, beside mesenteric malperfusion, coronary malperfusion also incorporates a high mortality risk. In comparison to this, cerebral malperfusion may lead to persistent neurologic deficits postoperatively. However, neurologic status is known to show an improvement after surgery regardless of age. Overall, the risk of mortality increases with the number of systems affected (43, 44).

Malperfusion can be managed in different ways (26). Either the approach is first central aortic repair in order to restore true lumen flow. Malperfusion is then expected to resolve in time. Another option would be endovascular treatment of the malperfusion first via separate branch stenting or fenestration and central aortic repair at a later point of time. The third option could be a hybrid approach, meaning the combination of endovascular treatment and open surgery in one session (7, 45). It is recognized that elderly patients who present ATAAD plus organ malperfusion (visceral, coronary, peripheral) form a particularly high-risk subgroup and that, in selected cases, deviating from the classic “central repair first” paradigm and using an initial (or hybrid) interventional strategy instead may improve outcomes in order to prevent malperfusion syndrome (45, 46). The 2024 ESC Guidelines for aortic dissection introduce new, more precise recommendations for surgical intervention, including a Class IIb recommendation for certain patient groups. Central repair remains urgent for unstable patients, but for stable patients with severe malperfusion, staged management is increasingly recommended (21). Modern management of malperfusion syndrome in aortic dissection emphasizes rapid diagnosis, organ-specific reperfusion, and individualized timing of central repair. By integrating symptom timing and organ viability into the decision-making process, this approach achieves significantly improved survival and a decrease in adverse events (7, 21, 43).

Malperfusion further determines the urgency of surgical intervention, since patients with critical end-organ ischemia have a higher mortality risk. This “symptom onset-to-cut time” may not be an independent risk factor for 30-day mortality in patients undergoing surgery for stable ATAAD and staying stable during the preoperative course (21). Immediate surgical intervention in stable ATAAD should be judiciously considered by an experienced aortic team, but rapid operation remains essential, and no compromises should be made if instability develops (21, 47, 48). Some centers perform a staged approach with “endovascular first” reperfusion by membrane fenestration or stenting of the affected branches prior to open repair in an attempt to stabilize the patient and reduce the time of end-organ ischemia (7). The decision “endovascular or central repair first”, as mentioned before, depends on the institutional experience, the availability of endovascular resources and the surgeon's know-how.

In this review 22.8% of patients showed preoperative malperfusion, highlighting the importance of innovative and alternative treatment concepts for these at-risk patients.

### Limited vs. extensive repair

The question of the extent of surgical treatment plays a pivotal part in the elderly undergoing surgery for ATAAD. The decision between reducing surgical trauma, operating times and circulatory arrest times in limited repair vs. reducing the risk of further dissection and avoiding the need for secondary interventions in extensive repair remains a challenge.

In this context, the standard of care hemiarch replacement assumes particular importance. As seen in this review, the distributions were favorable for hemiarch replacement, with total arch replacement in second place. The FET technique did not feature as a standard treatment option in the available literature and was referenced in only one study (17). This may be the preferred surgical attempt when preoperative malperfusion, frailty and comorbidities predominate (49). However, the risk of residual dissection and the further need for intervention must be borne in mind. In contrast, extensive repair would include total arch replacement with or without frozen elephant trunk implantation, both of which are associated with longer cross-clamp and circulatory arrest times and a higher perioperative risk for stroke and impairment of QoL in octogenarians post-surgery (50). Though these techniques significantly improve long-term outcomes and lower the risk for aortic-related re-interventions, they may be associated with a pronounced perioperative risk (51, 52). Given the fact that endovascular strategies for aortic repair are evolving and offer new approaches for aortic-related reinterventions, this approach may be justifiable considering the significantly increased operative risk of using FET in elderly patients.

### Endovascular and hybrid approaches

Open surgical repair remains the gold standard for ATAAD to guarantee excision of the primary entry tear in addition to replacing the ascending aorta (2, 53). However, hybrid and endovascular strategies have emerged as additional procedures or less invasive alternatives to complement the extent of repair, reduce surgical trauma and improve perioperative outcomes in specific patient cohorts (54, 55).

Various prostheses are available on the market to perform standardized thoracic endovascular aortic repair (TEVAR) of the descending thoracic aorta, e.g., GORE C-TAG® or TAG® (GORE®, Flagstaff, AZ, USA), ZENITH TX2 and Alpha (Cook®, Bloomington, IN USA), Valiant Captiva (Medtronic®, Dublin, Ireland) or Relay® (Bolton, Barcelona, Spain; now Terumo®, Inchinnan, UK) to name the most popular ones.

The hybrid approach refers to the combination of open surgical replacement of the ascending aorta and endovascular stent grafting of the distal aortic arch and descending thoracic aorta, performed either simultaneously or in a staged procedure. The most common hybrid techniques include the Frozen Elephant Trunk (FET) procedure, which utilizes a single prosthesis which is composed of a proximal surgical graft and a distal stent graft, and hemiarch replacement followed by TEVAR, applied either concomitantly or sequentially (54, 56). The latter strategy is particularly applicable in cases where distal malperfusion persists despite adequate proximal repair (57). Although the FET procedure is associated with an inherent risk of operative mortality and does not necessarily improve immediate perioperative survival, it provides significant mid- and long-term benefits. Furthermore, it promotes aortic remodeling and a substantial reduction in the need for subsequent aortic interventions. Therefore, while FET remains the most effective therapeutic option, its use in elderly patients should be approached with great caution and careful patient selection (51, 58).

The Ascyrus Medical Dissection Stent (AMDS; Artivion®, Atlanta, GA, USA) is a hybrid prosthesis designed as an adjunct to hemiarch repair in ATAAD, aiming to stabilize the true lumen, promote positive aortic remodeling and reduce malperfusion (see Supplementary Figure 5) (59). It was developed to upgrade the standard of care hemiarch procedure, aiming to reduce complications deriving from true lumen collapse and false lumen patency (60, 61). The stent consists of a proximal cuff composed of polytetrafluorethylene and an uncovered superhelical nitinol stent. The aim of the cuff is to effectively seal the distal anastomosis and prevent false lumen flow while lowering the risk for distal anastomotic new entries (DANEs) (59). Especially in elderly patients, the AMDS could represent an ideal solution and compromise in exactly these scenarios: For instance, for an 80-year-old patient with malperfusion and a high perioperative risk, undergoing an extensive procedure such as a FET may impose an unnecessary operative burden. A more limited approach consisting of hemiarch replacement combined with AMDS can shorten operating and circulatory arrest times while rapidly restoring true-lumen flow (59). However, this strategy should be reserved for patients with DeBakey type I dissection and is not appropriate when the primary entry tear is located in the aortic arch; in these situations, no compromise should be made and definitive arch repair remains mandatory. When appropriately selected, particularly in elderly and high-risk patients without an arch entry tear, this approach may provide a safer and more balanced alternative (61).

In contrast, endovascular repair of ATAAD involves the deployment of stent grafts in the ascending aorta without the need for open surgery. However, this approach is currently reserved for patients deemed unsuitable for conventional repair due to severe comorbidities (23). Since endovascular treatment requires precise proximal and distal landing zones as well as limited aortic diameters, it may not be anatomically feasible in all ATAAD cases (57). The number of devices that are designed for the ascending aorta (e.g., Zenith Ascend TAA Endovascular Graft or Cook Arch Branched Device by Cook Medical or RelayBranch Thoracic Stent-Graft System by Terumo Aortic) is scarce and presently undergoing early evaluation (62, 63). However, satisfactory results have been achieved by full endovascular treatment in selected cases deemed unsuitable for open ATAAD repair (23, 40). The ARISE study, for example, demonstrated that endovascular repair of ascending aortic dissection using a dedicated aortic stent graft is technically feasible in high-risk patients with the appropriate anatomy (64). These preliminary results support further investigation and a larger, pivotal study (ARISE II) has been initiated to evaluate the device in a larger cohort and possibly in broader indications beyond acute dissection. In the context of the ARISE III study evaluating the GORE® Ascending Stent Graft, which launched in December 2025, the first patient treated for acute Type A aortic dissection was successfully implanted with the stent graft. This study could establish the first endovascular stent graft option for treating acute type A dissections (64, 65). However, up to now open surgical repair remains the mandatory therapy for treating type A dissections.

In conclusion, endovascular and hybrid approaches may be considered for a carefully selected subset of elderly, frail patients with an excessive operative risk as well as those with a history of cardiac surgery, limited ascending aortic involvement or persistent malperfusion following proximal repair (62). Indeed, open repair remains primary choice as the first treatment modality in the majority of cases. However, in selected cases, elderly patients in particular may benefit significantly from alternative treatment concepts that reduce surgical trauma, operating and circulatory arrest times or even avoid standardized open repair (66). Another publication by Yang et al. showed that the 1.9% aorta-related mortality and 100% technical success rate are promising, especially considering these were high-risk patients, many with acute dissections (67).

An initial in-human application of a valve-integrated endovascular graft, called Endo-Bentall, has been described previously, although it was performed for a post-surgical pseudoaneurysm rather than a dissection. A more recent report published by Ghoreishi et al. described a series of 5 high-risk patients treated with a custom-made Endo-Bentall device between November 2022 and October 2023. Of those patients, 4 had acute type A dissection, 1 had an aortic root aneurysm. The outcome was very satisfactory, showing technical success in all patients as well as no in-hospital mortality or postoperative stroke. The follow-up after around 172 days showed no signs of type Ia endoleak as well as no aortic insufficiency on echocardiography. The Endo-Bentall procedure thus represents a minimally invasive approach to treat aortic root pathology in patients that are not feasible for surgery. Nonetheless, it remains, to date, a salvage or last-resort therapy reserved for carefully selected high-risk patients in specialized centers, given the need for custom-built grafts and the considerable technical complexity (68).

### Cerebral protection strategies and neurological outcome

Organ protection via hypothermia and cerebral perfusion play a crucial role during surgery of acute type A aortic dissection. Different types of strategies can be applied. Deep hypothermia in hypothermic circulatory arrest (DHCA) slows down cerebral metabolism but also shows a cumulative risk for stroke and an increased risk for delirium with lower degrees of hypothermia and shorter arrest times (47, 69). While deep hypothermia was the strategy of choice in the past, this approach is now outdated and no longer recommended by current guidelines (21, 70). Extended CPB and operating times, as well as prolonged ICU stays are established risk factors for POD (31). Hypothermia may contribute to this due to reduced cerebral blood flow or exacerbate hemodilution during CPB, which represents a compounding risk of cerebral hypoxia or micro-ischemia, both of which are known contributors to delirium (71). Selective antegrade cerebral perfusion (ACP) combined with moderate hypothermia allows sufficient perfusion and oxygenation of cerebral blood via axillary, innominate or carotid cannulation and lowers the risk for neurologic injury. This is the most commonly applied technique with positive outcomes in ATAAD (72). ACP can be administered unilaterally, bilaterally or even trilaterally. Clear clinical superiority of bilateral ACP has not been shown to date; therefore, the decision must be made individually and depends on patient factors such as anatomy as well as intraoperative factors, the expected extent of aortic repair and circulatory arrest duration. In comparison to this, retrograde cerebral perfusion (RCP) by venous perfusion during circulatory arrest aims at flushing out emboli retrogradely. Although ACP and RCP have shown a similar risk for intraoperative stroke, ACP is used more frequently as it represents the physiological flow and does not require deeper degrees of hypothermia (70). There is a lack of evidence as to whether deeper degrees of hypothermia have a significant impact on the incidence of delirium in elderly patients undergoing surgery for ATAAD, which may favor moderate or even mild hypothermia in this patient cohort (31, 32).

The decision regarding the cannulation method to be used should be based on anatomy and dissection patterns, while femoral cannulation should be avoided whenever possible (73–75). Regarding the cannulation strategy, axillary or central cannulation is generally preferred over femoral access to reduce the risk of malperfusion and perioperative stroke (76).

The challenge in ATAAD surgery remains in minimizing arrest time and optimizing perfusion management to prevent transient or permanent neurologic damage (77).

The use of ACP instead of DHCA alone can minimize cerebral ischemic time and has been associated with lower rates of stroke or transient neurological dysfunction (41).

Recent data suggest that in elderly or otherwise high-risk patients, a strategy of moderate hypothermic circulatory arrest (MHCA) rather than traditional DHCA may be advantageous (78). Historically, DHCA was thought to maximize neuroprotection in ATAAD surgery, yet contemporary evidence indicates that MHCA, especially with antegrade cerebral perfusion, can achieve similar organ protection while minimizing perioperative complications (79, 80).

### Quality of life post-surgery

Quality of life constitutes a pivotal consideration in surgery for ATAAD, as this event represents a profound turning point in patients' lives, with lasting implications for their physical, social and mental well-being. The assessment of QoL after aortic surgery has not been documented regularly.

Nevertheless, it has been shown that Health-related Quality of life (HR-QOL) declines after ATAAD surgery, mainly in categories such as physical functioning. Elderly patients are particularly vulnerable in physical domains, given the aspect of frailty.

In some studies, less use of DHCA or the use of ACP appeared to correlate with better HR-QOL outcomes (5).

Among older patients undergoing cardiac surgery, factors limiting recovery and possibly leading to negative long-term outcomes are physical frailty and comorbidity (81). However, many maintain or regain psychological well-being (82). In younger patients, on the other hand, perioperative inflammation and neurocognitive stress may transiently impair mental or neurocognitive function but baseline physical reserve often enables better functional recovery (83). The limited evidence suggests that overall postoperative quality of life may be satisfactory in many survivors. However, conclusions are restricted by the small number of studies and incoherent methods. The interpretation of these findings should be cautious, as quality of life outcomes were reported heterogenously with different follow-up intervals.

## Limitations

The included studies demonstrate heterogeneity in operative strategies for acute type A aortic dissection repair in patients older than 70 years. Therefore, pooled postoperative outcomes may have a limited interpretability and were reported descriptively and not stratified by intervention type.

Due to considerable clinical and methodological heterogeneity of the reviewed studies, it was not feasible to perform subgroup and sensitivity analyses in order to explore the possible source of heterogeneity. The available literature on ATAAD in the elderly patients was limited, particularly in regard to specific clinical and surgical aspects. As a result, the pooled estimates may not be applicable to all clinical settings. In order to improve the interpretation of clinical outcomes, future research should apply standardized reporting methods and focus on more homogenous patient cohorts, which would then allow a more precise assessment of the variables of interest.

In terms of malperfusion, it has been identified as an important prognostic factor. However, the included studies provided incoherent information on what approach was performed regarding management strategies. Consequently, a systematic comparison of treatment approaches was not possible.

The aforementioned variables are crucial and known to influence perioperative risk as well as clinical outcome. In order to improve the interpretation of the latter, future research should apply standardized reporting methods and focus on more homogenous patient cohorts, which would then allow a more precise assessment of the variables of interest.

Although this systematic review was not prospectively registered, the review process followed a predefined methodology and was conducted in accordance with PRISMA guidelines. We further did nor consider its absence to have influenced the succeeding work.

In summary, several limitations must be acknowledged. First, all included studies were observational, mostly in a retrospective design which may lead to selection and reporting bias. Second, studies could not have been compared easily due to their heterogeneity in operative strategies and patient selection. Third, management of malperfusion was inconsistently reported, highlighting the importance of subgroup analyses. Finally, data on long-term outcomes and quality of life were limited and reduced on only a small number of studies.

## Conclusions

The surgical management of ATAAD in elderly patients remains a formidable challenge due to age-related frailty, comorbidities and an increased susceptibility to organ dysfunction. Mortality and morbidity remain higher than in younger cohorts, although advances in operative strategies, cerebral and organ protection as well as perioperative management have been made. Therefore, surgical repair of ATAAD should be carefully considered and preoperatively assessed including an evaluation of frailty, malperfusion and overall physiological reserve in order to determine the optimal treatment strategy. This concept is ideally managed in specialized aortic centers by experienced multidisciplinary teams capable of offering combined surgical and innovative endovascular approaches. Keeping this in mind, the burden of residual dissection and late complications must be balanced against primary life-saving limited surgical repair strategies. With the development of hybrid and endovascular techniques, high-risk or even inoperable elderly patients may be candidates for specific treatment strategies. However, these remain investigational and are limited by technical, anatomical and expertise-related barriers. Ultimately, open surgical repair continues to represent the gold standard of ATAAD management, providing the most definitive treatment in most cases.

Ongoing innovations in organ protection, perioperative optimization and minimally invasive techniques are expected to further enhance survival and postoperative quality of life in elderly patients with ATAAD. However, surgery in septuagenarians and octogenarians without malperfusion can yield satisfactory outcomes, indicating that advanced age alone should not be regarded as a contraindication to surgical management *per se*.

By integrating data from studies from the last 20 years, we provide an overview regarding current surgical treatment of ATAAD in the elderly. The balance between operative risk and potential survival benefit in this increasingly relevant patient cohort is being highlighted and critically discussed in this review.

## Data Availability

The original contributions presented in the study are included in the article/supplementary material, further inquiries can be directed to the corresponding author/s.
