# Supplementary material for: Surgical strategies in elderly patients with acute type A aortic dissection—a systematic review
Source: Front Surg. 2026 Jul 24;13:1856125. doi: 10.3389/fsurg.2026.1856125 (PMC13449147; doi:10.3389/fsurg.2026.1856125)
Supplement: Supplementary file 1 [file Supplementaryfile1.docx]

**Supplementary materials**

**TEVAR: Thoracic Endovascular Aortic Repair, FET: Frozen Elephant Trunc, AMDS: Ascyrus Medical Dissection Stent, DANE:distal anatomic new entries, DHCA: Deep Hypothermic Circulatory Arrest, ACP: Antegrade Cerebral Perfusion, MHCA: Moderate Hypothermic Circulatory Arrest*

**Figure 1** Graphical Abstract: Main aspects in ATAAD surgery in the elderly: endovascular and hybrid approaches, limited vd. Extensive repair, cerebral protection & neurological outcome and malperfusion. For further information see discussion.

**Identification of studies via databases**

Records removed *before screening*:

Duplicate records removed

(n = 904)

1. PubMed (n=12)
2. Ovid/Medline (n=798)
3. Web of Science (n=94)
4. By hand (n=12)

Records identified (n = 1,910) from:

1. PubMed (n = 879)
2. Ovid/Medline (n = 811)
3. Web of Science (n = 203)
4. By hand (n = 17)

**Identification**

Records excluded

(n = 917)

- Age >70 years only
- Mixed-age cohorts where patients >70 cannot be extracted
- Studies on type B aortic dissection only
- Others

Records screened for Title/Abstract

(n = 1,006)

**Screening**

Reports not retrieved

(n = 33)

Reason 1: not relevant

(n = 18)

Reason 2: no full (English) text available

(n = 15)

Reports sought for retrieval

(n = 89)

Reports excluded

(n = 15)

Reason: >3 criteria missing for specific age/indication/management group

(n = 15)

**Included**

Reports assessed for eligibility

(n = 56)

Studies included in review

(n = 41)

- ***MeSH Terms and Boolean operators***
  a. **Pubmed**: („Type A Aortic Dissection*”[All Fields] AND “Aged”[MeSH Terms] AND “Outcome*”[All Fields]) NOT “Type B Aortic Dissection*”[All Fields]
  b. **Ovid/Medline:** (“Type A Aortic Dissection* “.af. and exp Aged/ and Outcome*.af.) not “Type B Aortic Dissection*”.af.
  c. **Web of Science:** (ALL=”Type A Aortic Dissection*” AND (ALL=Aged OR ALL=Elderly OR ALL=Octogenarian* OR ALL=Septuagenarian* OR ALL=Nonagenarian* OR ALL=Old) AND ALL=Outcome*) NOT ALL=”Type B Aortic Dissection*”

**Figure 2.** PRISMA (Preferred Reporting Items for Systematic Review and Meta-Analysis) flowchart of the systematic literature review from Page MJ, McKenzie JE, Bossuyt PM, Boutron I, Hoffmann TC, Mulrow CD, et al. The PRISMA 20ƒstatement: an updated guideline for reporting systematic reviews. BMJ 2021;372:n71. doi: 10.1136/bmj.n71.

Number of publications

|  | No. of publications |
| --- | --- |
| 2001 | 1 |
| 2002 | 1 |
| 2006 | 2 |
| 2008 | 1 |
| 2009 | 2 |
| 2010 | 2 |
| 2011 | 1 |
| 2013 | 4 |
| 2014 | 1 |
| 2015 | 4 |
| 2016 | 2 |
| 2017 | 3 |
| 2019 | 2 |
| 2020 | 1 |
| 2021 | 2 |
| 2022 | 6 |
| 2023 | 4 |
| 2024 | 2 |
| 2025 | 4 |

**Figure 3.** Number of included publications per year; included were publications from 2001-2025.

**Figure 4.** (A) Preoperative computed tomography-based multiplanar 3D reconstruction of the full range of the aorta, and (B) centerline measurement

**Figure 5.** Postoperative computed tomography-based 3D reconstruction of the aorta with implanted AMDS device (Artivion®, Atlanta, GA, USA).

**Table 1:** List of reviewed publications

|  | **author** | **year** | **region** | **N** | **DOI** | **Title** | **Study design** | **Journal** |
| --- | --- | --- | --- | --- | --- | --- | --- | --- |
| 1 | Ahmad et al. | 2015 | Germany | 39 | [DOI: 10.1016/j.athoracsur.2014.08.020](https://doi.org/10.1016/j.athoracsur.2014.08.020) | *Surgical repair for acute type A aortic dissection in octogenarians* | Evaluation study | ANNALS OF THORACIC SURGERY |
| 2 | Berndt et al. | 2015 | Germany | 65 | [DOI: 10.1055/s-0034-1395985](https://doi.org/10.1055/s-0034-1395985) | *Outcome after surgery for acute aortic dissection type A in the elderly: a single-center experience* | Comparative study | Thorac Cardiovasc Surg |
| 3 | Bojko et al. | 2022 | USA | 165 | [DOI: 10.1016/j.jtcvs.2020.03.157](https://doi.org/10.1016/j.jtcvs.2020.03.157) | *Midterm outcomes of emergency surgery for acute type A aortic dissection in octogenarians* | Retrospective study | J Thorac Cardiovasc Surg, |
| 4 | Caus et al. | 2002 | France | 83 | [DOI: 10.1016/s1010-7940(02)00275-0](https://doi.org/10.1016/s1010-7940(02)00275-0) | *Clinical outcome after repair of acute type A dissection in patients over 70 years-old* | Retrospective Study | Eur J Cardiothorac Surg |
| 5 | Chen et al. | 2022 | Taiwan | 123 | [DOI: 10.1111/jocs.16219](https://doi.org/10.1111/jocs.16219) | *Outcome of emergency surgery for acute type A aortic dissection in octogenarians* | Retrospective Study | Journal of cardiac surgery |
| 6 | Chen et al. | 2023 | China | 1,092 | [DOI: 10.21037/cdt-22-533](https://doi.org/10.21037/cdt-22-533) | *Safety of open limited surgery for septuagenarian and octogenarian acute type A aortic dissection patients: a retrospective cohort study.* | Retrospective observational cohort study | Cardiovasc Diagn Ther |
| 7 | Dong et al. | 2023 | China | 640 | [DOI: 10.1186/s12872-023-03513-3](https://doi.org/10.1186/s12872-023-03513-3) | *Early outcomes of a triple-branched stent graft implantation in elderly patients with acute type a aortic dissection.* | Retrospective observational cohort study | BMC Cardiovasc Disord, |
| 8 | Dumfarth et al. | 2017 | Austria | 90 | [DOI: 10.1093/ejcts/ezw387](https://doi.org/10.1093/ejcts/ezw387) | *Acute type A dissection in octogenarians: does emergency surgery impact in-hospital outcome or long-term survival?* E | Multicenter Study | Eur J Cardiothorac Surg, |
| 9 | Hata et al. | 2010 | Japan | 27 | [DOI: 10.1016/j.athoracsur.2010.01.050](https://doi.org/10.1016/j.athoracsur.2010.01.050) | *Midterm outcomes of rapid, minimally invasive resection of acute type A aortic dissection in octogenarians.* | Retrospective Case Series | Ann Thorac Surg |
| 10 | Hattori et al. | 2020 | Japan | 273 | [DOI: 10.1093/icvts/ivaa061](https://doi.org/10.1093/icvts/ivaa061) | *Acute type A aortic dissection in non-agenarians: to cut or not.* | Multicenter Study | Interact Cardiovasc Thorac Surg |
| 11 | Jussli-Melchers et al. | 2017 | Germany | 242 | [DOI: 10.1093/ejcts/ezw408](https://doi.org/10.1093/ejcts/ezw408) | *Long-term outcome and quality of life following emergency surgery for acute aortic dissection type A: a comparison between young and elderly adults.* | Comparative Study | Eur J Cardiothorac Surg |
| 12 | Kageyama et al. | 2024 | Japan | 1,037 | [DOI: 10.1016/j.jtcvs.2022.02.019](https://doi.org/10.1016/j.jtcvs.2022.02.019) | *Early mortality of emergency surgery for acute type A aortic dissection in octogenarians and nonagenarians: A multi-center retrospective study* | Multicenter Study | J Thorac Cardiovasc Surg |
| 13 | Kilic et al. | 2013 | USA | 117 | [DOI: 10.1093/icvts/ivt155](https://doi.org/10.1093/icvts/ivt155) | *Outcomes in the current surgical era following operative repair of acute Type A aortic dissection in the elderly: a single-institutional experience* | Retrospective cohort study | Interact Cardiovasc Thorac Surg, |
| 14 | Komatsu et al. | 2014 | Japan | 59 | [DOI: 10.1016/j.athoracsur.2014.01.045](https://doi.org/10.1016/j.athoracsur.2014.01.045) | *Surgical outcomes of acute type A aortic dissection in elderly patients* | Evaluation Study | Ann Thorac Surg |
| 15 | Kondo et al. | 2019 | Japan | 70 | [DOI: 10.1007/s11748-019-01111-9](https://doi.org/10.1007/s11748-019-01111-9) | *Treatment outcomes for acute type A aortic dissection with patent false lumen in patients over the age of 80.* | Retrospective cohort study | Gen Thorac Cardiovasc Surg |
| 16 | Salem et al. | 2021 | Germany | 368 | [DOI: 10.3390/jcm10081643](https://doi.org/10.3390/jcm10081643) | *Influence of Age on Postoperative Neurological Outcomes after Surgery of Acute Type A Aortic Dissection.* | Retrospective comparative cohort study | JOURNAL OF CLINICAL MEDICINE |
| 17 | Kondoh et al. | 2019 | Japan | 436 | [DOI: 10.1016/j.jtcvs.2016.03.093](https://doi.org/10.1016/j.jtcvs.2016.03.093) | *Outcomes of limited proximal aortic replacement for type A aortic dissection in octogenarians.* | Retrospective observational cohort study | J Thorac Cardiovasc Surg |
| 18 | Malvindi et al. | 2015 | UK | 45 | [DOI: 10.1093/ejcts/ezu543](https://doi.org/10.1093/ejcts/ezu543) | *Acute type A aortic dissection repair in elderly patients.* | Retrospective observational cohort study | Eur J Cardiothorac Surg |
| 19 | Maze et al. | 2022 | Japan | 174 | [DOI: 10.1186/s13019-022-01819-5](https://doi.org/10.1186/s13019-022-01819-5) | *Clinical outcomes of limited repair and conservative approaches in older patients with acute type A aortic dissection.* | Retrospective cohort study | J Cardiothorac Surg |
| 20 | Morjan et al. | 2022 | Switzerland | 638 | [DOI: 10.21037/jtd-21-1863](https://doi.org/10.21037/jtd-21-1863) | *The impact of age and sex on in hospital outcomes in acute type A aortic dissection surgery.* J | Retrospective cohort study | JOURNAL OF THORACIC DISEASE |
| 21 | Nakai et al. | 2022 | Japan | 254 | [DOI: 10.1177/02184923221083369](https://doi.org/10.1177/02184923221083369) | *Surgical outcomes of acute type A aortic dissection in septuagenarians and octogenarians* | Retrospective cohort study | Asian Cardiovasc Thorac Ann, |
| 22 | Neri et al. | 2001 | Italy | 24 | [DOI: 10.1067/mtc.2001.112205](https://doi.org/10.1067/mtc.2001.112205) | *Operation for acute type A aortic dissection in octogenarians: is it justified?* | Retrospective Case Series | J Thorac Cardiovasc Surg, |
| 23 | Omura et al. | 2017 | Japan | 345 | [DOI: 10.1016/j.athoracsur.2016.05.046](https://doi.org/10.1016/j.athoracsur.2016.05.046) | *Early and Late Outcomes of Operation for Acute Type A Aortic Dissection in Patients Aged 80 Years and Older* | Retrospective cohort study | Ann Thorac Surg, |
| 24 | Park et al. | 2024 | Korea | 471 | [DOI: 10.1093/icvts/ivae038](https://doi.org/10.1093/icvts/ivae038) | *Acute type A aortic dissection features and outcomes in octogenarians: a propensity score analysis* | Retrospective cohort study with Propensity Score Matching | Interdiscip Cardiovasc Thorac Surg, |
| 25 | Piccardo et al. | 2009 | France | 57 | [DOI: 10.1016/j.athoracsur.2009.04.096](https://doi.org/10.1016/j.athoracsur.2009.04.096) | *Outcomes after surgical treatment for type A acute aortic dissection in octogenarians: a multicenter study.* | Multicenter Study | Ann Thorac Surg |
| 26 | Piccardo et al. | 2009 | France | 319 | not available | *Should octogenarians be denied access to surgery for acute type A aortic dissection?* | Retrospective single-Center Case Series | J Cardiovasc Surg (Torino) |
| 27 | Piccardo et al. | 2013 | France | 79 | [DOI: 10.1016/j.athoracsur.2013.04.066](https://doi.org/10.1016/j.athoracsur.2013.04.066) | *Octogenarians with uncomplicated acute type a aortic dissection benefit from emergency operation* | Comparative Study | Ann Thorac Surg, |
| 28 | Pitts et al. | 2023 | Germany | 204 | [DOI: 10.1093/ejcts/ezad288](https://doi.org/10.1093/ejcts/ezad288) | *The Impact of Malperfusion Patterns in Elderly Patients undergoing Surgery for Acute Type A Aortic Dissection.* | Retrospective observational cohort study | Eur J Cardiothorac Surg, |
| 29 | Rylski et al. | 2011 | Germany | 1,558 | [DOI: 10.1016/j.ejcts.2010.11.073](https://doi.org/10.1016/j.ejcts.2010.11.073) | *Outcome after surgery for acute aortic dissection type A in patients over 70 years: data analysis from the German Registry for Acute Aortic Dissection Type A (GERAADA).* | Multicenter Study | Eur J Cardiothorac Surg |
| 30 | Santini et al. | 2006 | Italy | 247 | [DOI: 10.1016/j.ejcts.2005.12.016](https://doi.org/10.1016/j.ejcts.2005.12.016) | *Survival and quality of life after repair of acute type A aortic dissection in patients aged 75 years and older justify intervention* | Evaluation Study | Eur J Cardiothorac Surg |
| 31 | Shiono et al. | 2006 | Japan | 134 | [DOI: 10.1016/j.athoracsur.2005.12.048](https://doi.org/10.1016/j.athoracsur.2005.12.048) | *Emergency surgery for acute type a aortic dissection in octogenarians.* | Retrospective Case Series | Ann Thorac Surg |
| 32 | Shrestha et al. | 2008 | Germany | 24 | [DOI: 10.1177/021849230801600109](https://doi.org/10.1177/021849230801600109) | *Is treatment of acute type A aortic dissection in septuagenarians justifiable?* | Retrospective single-center case series | Asian Cardiovasc Thorac Ann, |
| 33 | Stamou et al. | 2010 | USA | 119 | [DOI: 10.1510/icvts.2009.222984](https://doi.org/10.1510/icvts.2009.222984) | *Is advanced age a contraindication for emergent repair of acute type A aortic dissection?* | Comparative Study | Interact Cardiovasc Thorac Surg |
| 34 | Suenaga et al. | 2016 | Japan | 117 | [DOI: 10.1007/s11748-015-0613-0](https://doi.org/10.1007/s11748-015-0613-0) | *Ascending aortic replacement for acute type A aortic dissection in octogenarians.* | Retrospective Cohort study | Gen Thorac Cardiovasc Surg, |
| 35 | Tang et al. | 2013 | USA | 101 | [DOI: 10.1016/j.jtcvs.2012.11.060](https://doi.org/10.1016/j.jtcvs.2012.11.060) | *Surgery for acute type A aortic dissection in octogenarians is justified.* | Comparative Study | J Thorac Cardiovasc Surg, |
| 36 | Tashima et al. | 2021 | Japan | 103 | [DOI: 10.1111/jocs.15617](https://doi.org/10.1111/jocs.15617) | *Physical activities and surgical outcomes in elderly patients with acute type A aortic dissection* | Retrospective Cohort Study | *.* J Card Surg |
| 37 | Tochii et al. | 2016 | Japan | 158 | [DOI: 10.1253/circj.CJ-16-0918](https://doi.org/10.1253/circj.cj-16-0918) | *Early and Late Outcomes of Surgical Repair for Stanford A Acute Aortic Dissection in Octogenarians.* | Comparative Study | Circ J |
| 38 | Wang et al. | 2022 | China | 1,092 | [DOI: 10.1186/s13019-022-01785-y](https://doi.org/10.1186/s13019-022-01785-y) | *The impact of age in acute type A aortic dissection: a retrospective study* | Retrospective Cohort Study | J Cardiothorac Surg |
| 39 | Zhang et al. | 2023 | China | 714 | [DOI: 10.1097/JS9.0000000000000342](https://doi.org/10.1097/js9.0000000000000342) | *Septuagenarians with acute type A aortic dissection undergoing extended aortic arch repair: a retrospective cohort study* | Retrospective Cohort Study with Propensity Score Matching | Int J Surg, |
| 40 | Zheng et al. | 2013 | China | 11 | DOI: 10.1186/1749-8090-8-78 | *Surgical management for acute type A aortic dissection in patients over 70 years-old.* | Retrospective single-center case series | JOURNAL OF CARDIOTHORACIC SURGERY, |
| 41 | Zindovic et al. | 2015 | Sweden | 341 | [DOI: 10.1111/jocs.12633](https://doi.org/10.1111/jocs.12633) | *Impact of Hemodynamic Instability and Organ Malperfusion in Elderly Surgical Patients Treated for Acute Type A Aortic Dissection* | Retrospective Cohort Srudy | J Card Surg |

**Table 2a:** Data extraction; IMH: intramural haematoma, BMI: body mass index

| **Author** | **N** | **age** | **pre shock** | **pre malperfusion** | **myocardial** | **limb** | **renal** | **cerebral** | **visceral** | **bmi** | **female** | **male** | **EuroSCORE II** | **DeBakey I** | **DeBakey II** | **imh** |
| --- | --- | --- | --- | --- | --- | --- | --- | --- | --- | --- | --- | --- | --- | --- | --- | --- |
| 1 | 39,00 | 82,00 | N/A | 7,00 | N/A | N/A | N/A | 7,00 | N/A | N/A | N/A | N/A | N/A | N/A | N/A | N/A |
| 2 | 65,00 | 74,00 | 16,00 | N/A | 2,00 | 8,00 | 6,00 | N/A | N/A | N/A | N/A | N/A | N/A | N/A | N/A | N/A |
| 3 | 165,00 | 74,00 | 30,00 | 42,00 | N/A | N/A | N/A | N/A | N/A | 27,41 | 81,00 | N/A | N/A | N/A | N/A | N/A |
|  | 70,00 | 83,50 | 19,00 | 11,00 | N/A | N/A | N/A | N/A | N/A | 26,15 | 42,00 | N/A | N/A | N/A | N/A | N/A |
| 4 | 41,00 | 74,80 | 29,00 | N/A | N/A | 7,00 | 3,00 | 10,00 | 7,00 | N/A | 16,00 | N/A | N/A | N/A | N/A | N/A |
|  | 42,00 | 75,60 | 0,00 | 0,00 | N/A | N/A | N/A | N/A | N/A | N/A | 18,00 | N/A | N/A | N/A | N/A | N/A |
|  | 31,00 | N/A | 16,00 | N/A | 5,00 | N/A | N/A | 8,00 | 7,00 | N/A | 11,00 | N/A | N/A | N/A | N/A | N/A |
|  | 52,00 | N/A | 12,00 | N/A | 8,00 | N/A | N/A | 5,00 | N/A | N/A | 23,00 | N/A | N/A | N/A | N/A | N/A |
|  | 47,00 | N/A | N/A | N/A | N/A | N/A | N/A | N/A | N/A | N/A | N/A | N/A | N/A | N/A | N/A | N/A |
|  | 36,00 | N/A | N/A | N/A | N/A | N/A | N/A | N/A | N/A | N/A | N/A | N/A | N/A | N/A | N/A | N/A |
| 5 | 18,00 | 82,00 | 7,00 | 0,00 | N/A | N/A | N/A | N/A | N/A | N/A | N/A | N/A | N/A | 15,00 | 3,00 | 5,00 |
| 6 | 136,00 | 75,00 | 9,00 | 42,00 | N/A | N/A | N/A | N/A | N/A | N/A | N/A | N/A | N/A | N/A | N/A | N/A |
|  | 86,00 | 75,00 | 19,00 | N/A | N/A | N/A | N/A | N/A | N/A | 24,40 | N/A | 40,00 | N/A | N/A | N/A | N/A |
|  | 50,00 | 74,50 | 4,00 | N/A | N/A | N/A | N/A | N/A | N/A | 24,00 | N/A | 34,00 | N/A | N/A | N/A | N/A |
| 7 | 67,00 | 73,50 | N/A | N/A | 3,00 | 4,00 | 6,00 | 3,00 | N/A | N/A | N/A | 45,00 | N/A | N/A | N/A | N/A |
| 8 | 67,00 | 83,00 | N/A | 11,00 | N/A | N/A | N/A | N/A | N/A | 26,10 | N/A | 33,00 | N/A | 36,00 | 31,00 | N/A |
| 9 | 27,00 | 81,70 | 1,00 | N/A | N/A | N/A | N/A | N/A | N/A | N/A | 19,00 | N/A | N/A | N/A | N/A | 12,00 |
| 10 | 10,00 | 92,00 | 2,00 | 1,00 | N/A | N/A | N/A | N/A | N/A | N/A | N/A | 2,00 | N/A | 5,00 | 4,00 | N/A |
| 11 | 78,00 | 76,00 | 20,00 | N/A | 2,00 | 10,00 | 7,00 | N/A | N/A | 26,00 | N/A | 38,00 | N/A | 5,00 | 66,00 | N/A |
| 12 | 227,00 | 83,80 | 78,00 | N/A | 10,00 | 7,00 | N/A | 46,00 | 3,00 | N/A | N/A | N/A | N/A | N/A | N/A | N/A |
| 13 | 31,00 | 78,00 | N/A | 6,00 | N/A | N/A | N/A | N/A | N/A | 29,50 | 11,00 | 20,00 | N/A | N/A | N/A | N/A |
| 14 | 59,00 | 77,00 | 18,00 | 16,00 | N/A | N/A | N/A | N/A | N/A | N/A | N/A | N/A | N/A | N/A | N/A | N/A |
|  | 51,00 | 77,40 | 14,00 | 13,00 | N/A | N/A | N/A | N/A | N/A | N/A | N/A | N/A | N/A | N/A | N/A | N/A |
|  | 8,00 | 74,80 | 4,00 | 3,00 | N/A | N/A | N/A | N/A | N/A | N/A | N/A | N/A | N/A | N/A | N/A | N/A |
|  | 15,00 | N/A | N/A | N/A | N/A | N/A | N/A | N/A | N/A | N/A | N/A | N/A | N/A | N/A | N/A | N/A |
|  | 44,00 | N/A | N/A | N/A | N/A | N/A | N/A | N/A | N/A | N/A | N/A | N/A | N/A | N/A | N/A | N/A |
| 15 | 44,00 | 83,50 | N/A | N/A | 2,00 | 3,00 | N/A | 7,00 | 2,00 | N/A | N/A | 12,00 | N/A | 30,00 | 10,00 | N/A |
| 16 | 68,00 | 79,00 | 8,00 | N/A | N/A | N/A | N/A | N/A | N/A | N/A | 40,00 | N/A | 9,60 | N/A | N/A | N/A |
| 17 | 77,00 | 83,00 | 9,00 | 2,00 | N/A | N/A | N/A | N/A | N/A | 20,80 | 59,00 | 18,00 | 42,30 | 60,00 | 17,00 | N/A |
|  | 73,00 | N/A | N/A | N/A | N/A | N/A | N/A | N/A | N/A | N/A | N/A | N/A | N/A | N/A | N/A | N/A |
|  | 4,00 | N/A | N/A | N/A | N/A | N/A | N/A | N/A | N/A | N/A | N/A | N/A | N/A | N/A | N/A | N/A |
| 18 | 45,00 | 79,00 | N/A | N/A | 5,00 | N/A | 2,00 | 5,00 | N/A | N/A | 26,00 | N/A | N/A | N/A | 3,00 | N/A |
| 19 | 31,00 | 83,90 | 8,00 | 6,00 | N/A | N/A | N/A | N/A | N/A | N/A | N/A | 10,00 | N/A | 16,00 | 15,00 | N/A |
| 20 | 143,00 | 80,60 | N/A | 86,00 | N/A | N/A | N/A | N/A | N/A | N/A | 68,00 | N/A | N/A | N/A | N/A | N/A |
|  | 75,00 | N/A | N/A | N/A | N/A | N/A | N/A | N/A | N/A | N/A | N/A | 75,00 | N/A | N/A | N/A | N/A |
|  | 68,00 | N/A | N/A | N/A | N/A | N/A | N/A | N/A | N/A | N/A | 68,00 | N/A | N/A | N/A | N/A | N/A |
| 21 | 59,00 | 75,00 | 21,00 | 20,00 | N/A | N/A | N/A | N/A | N/A | N/A | N/A | N/A | N/A | N/A | N/A | N/A |
|  | 32,00 | 84,50 | 14,00 | 11,00 | N/A | N/A | N/A | N/A | N/A | N/A | N/A | N/A | N/A | N/A | N/A | N/A |
| 22 | 24,00 | 83,00 | N/A | N/A | 8,00 | N/A | 15,00 | 10,00 | N/A | N/A | 7,00 | N/A | N/A | N/A | N/A | N/A |
| 23 | 63,00 | 83,70 | 14,00 | 12,00 | N/A | N/A | N/A | N/A | N/A | N/A | N/A | 28,00 | N/A | N/A | N/A | N/A |
| 24 | 48,00 | 83,00 | N/A | N/A | 0,00 | 2,00 | 2,00 | 5,00 | 2,00 | 22,40 | N/A | 7,00 | N/A | N/A | 20,00 | 19,00 |
| 25 | 57,00 | 82,20 | 13,00 | 25,00 | N/A | N/A | N/A | N/A | N/A | 30,00 | N/A | 30,00 | N/A | N/A | N/A | N/A |
| 26 | 57,00 | 82,20 | 7,00 | N/A | N/A | N/A | N/A | N/A | N/A | N/A | N/A | N/A | N/A | N/A | N/A | N/A |
| 27 | 23,00 | 81,60 | 20,00 | N/A | N/A | N/A | N/A | 12,00 | 4,00 | N/A | N/A | N/A | N/A | N/A | N/A | N/A |
| 28 | 400,00 | 76,00 | 95,00 | 174,00 | N/A | N/A | N/A | N/A | N/A | N/A | 189,00 | N/A | N/A | N/A | N/A | N/A |
| 29 | 381,00 | 73,70 | N/A | N/A | N/A | N/A | N/A | 30,50 | N/A | N/A | N/A | 184,00 | N/A | N/A | N/A | N/A |
|  | 83,00 | 82,80 | N/A | N/A | N/A | N/A | N/A | 7,50 | N/A | N/A | N/A | 26,00 | N/A | N/A | N/A | N/A |
| 30 | 40,00 | 78,00 | 9,00 | N/A | 1,00 | 11,00 | 14,00 | 3,00 | N/A | N/A | N/A | 21,00 | N/A | N/A | N/A | N/A |
| 31 | 24,00 | 82,00 | N/A | N/A | 1,00 | 1,00 |  | 8,00 | 1,00 | N/A | 14,00 | N/A | N/A | 16,00 | 7,00 | N/A |
| 32 | 24,00 | 75,00 | N/A | N/A | N/A | N/A | N/A | 6,00 | N/A | N/A | N/A | N/A | N/A | N/A | N/A | N/A |
| 33 | 29,00 | 77,00 | 2,32 | N/A | N/A | N/A | N/A | N/A | N/A | N/A | 15,08 | N/A | N/A | N/A | N/A | N/A |
| 34 | 25,00 | 83,90 | 8,00 | 6,00 | N/A | N/A | N/A | N/A | N/A | N/A | N/A | 6,00 | N/A | 9,00 | N/A | N/A |
| 35 | 21,00 | 85,00 | 2,00 | 1,00 | N/A | N/A | N/A | N/A | N/A | N/A | 17,00 | N/A | N/A | N/A | N/A | N/A |
| 36 | 52,00 | 78,00 | 11,00 | 7,00 | N/A | N/A | N/A | N/A | N/A | 23,10 | N/A | 11,00 | N/A | 35,00 | 17,00 | N/A |
| 37 | 24,00 | 83,00 | 4,00 | 0,00 | N/A | N/A | N/A | N/A | N/A | N/A | 19,00 | N/A | N/A | N/A | 9,00 | N/A |
| 38 | 118,00 | 74,00 | 6,00 | N/A | 5,00 | 12,00 | N/A | 19,00 | 7,00 | 24,10 | N/A | 64,00 | N/A | N/A | N/A | N/A |
|  | 18,00 | 82,50 | 3,00 | N/A | 0,00 | 1,00 | N/A | 1,00 | 0,00 | 23,40 | N/A | 10,00 | N/A | N/A | N/A | N/A |
| 39 | 65,00 | 71,00 | N/A | N/A | 2,00 | 2,00 | N/A | 7,00 | 6,00 | N/A | 33,00 |  | 9,00 | N/A | N/A | N/A |
| 40 | 11,00 | 72,20 | 0,00 | N/A | N/A | N/A | N/A | N/A | N/A | N/A | N/A | N/A | N/A | N/A | N/A | N/A |
| 41 | 101,00 | 75,30 | N/A | 47,00 | N/A | N/A | N/A | N/A | N/A | N/A | N/A | N/A | N/A | N/A | N/A | N/A |
| Mean |  | 79,22 | 14,93 | 21,96 | 3,60 | 5,67 | 6,88 | 10,53 | 3,90 | 25,18 | N/A | N/A | 20,30 | 22,70 | 16,83 | 12,00 |
| SD |  | 4,38 | 18,79 | 36,62 | 3,01 | 3,84 | 4,75 | 10,53 | 2,55 | 2,59 | N/A | N/A | 15,56 | 16,54 | 16,79 | 5,72 |
| ∑ | 3969,00 |  |  |  | 54,00 | 68,00 | 55,00 | 200,00 | 39,00 |  | 776,08 | 714,00 |  | 227,00 | 202,00 | 36,00 |

**Table 2b:** Data extraction; bav= bicuspid aortic valve, cad= coronary artery disease, pad= peripheral arterial disease, av= aortic valve, ah= arterial hypertension, copd= Chronic obstructive pulmonary disease

| **Author** | **bav** | **marfan** | **acute type a** | **rescuscitation** | **rupture** | **tamponade** | **cad** | **pad** | **av regurgitation more 2** | **av stenosis** | **ah** | **smoking** | **alcohol** | **diabetes** | **pre neurologic dysfunction** | **copd** |
| --- | --- | --- | --- | --- | --- | --- | --- | --- | --- | --- | --- | --- | --- | --- | --- | --- |
| 1 | N/A | N/A | 39,00 | N/A | N/A | N/A | 15,00 | N/A | 12,00 | 4,00 | 28,00 | N/A | N/A | N/A | 7,00 | 21,00 |
| 2 | N/A | N/A | N/A | N/A | N/A | N/A | N/A | N/A | N/A | N/A | N/A | N/A | N/A | N/A | N/A |  |
| 3 | 8,00 | 2,00 | N/A | 8,00 | 46,00 | N/A | N/A | 63,00 | 40,00 | N/A | 150,00 | 39,00 | N/A | 34,00 | N/A | 34,00 |
|  | 0,00 | 0,00 | N/A | 7,00 | 29,00 | N/A | N/A | 27,00 | 21,00 | N/A | 63,00 | 12,00 | N/A | 4,00 | N/A | 10,00 |
| 4 | N/A | 0,00 | N/A | N/A | N/A | N/A | N/A | 6,00 | N/A | N/A | 25,00 | N/A | N/A | N/A | N/A | 3,00 |
|  | N/A | 1,00 | N/A | N/A | N/A | N/A | N/A | 8,00 | N/A | N/A | 25,00 | N/A | N/A | N/A | N/A | 4,00 |
|  | N/A | N/A | N/A | 2,00 | N/A | N/A | N/A | N/A | N/A | N/A | N/A | N/A | N/A | N/A | N/A | N/A |
|  | N/A | N/A | N/A | 0,00 | N/A | N/A | N/A | N/A | N/A | N/A | N/A | N/A | N/A | N/A | N/A | N/A |
| 5 | 0,00 | 0,00 | N/A | N/A | N/A | N/A | N/A | N/A | N/A | N/A | N/A | N/A | N/A | N/A | N/A | N/A |
| 6 |  |  | N/A |  |  |  |  |  |  |  |  |  |  |  |  |  |
|  |  |  |  |  |  |  | 4,00 |  |  |  | 67,00 | 13,00 | 9,00 | 7,00 |  | 3,00 |
|  | N/A | N/A | N/A | N/A | N/A | N/A | 4,00 | N/A | N/A | N/A | 37,00 | 5,00 | 4,00 | 2,00 | N/A | 1,00 |
| 7 | N/A | 0,00 | N/A | N/A | N/A | N/A | N/A | 6,00 | 12,00 | N/A | 59,00 | N/A | N/A | 11,00 | N/A | 7,00 |
| 8 | N/A | N/A | N/A | 5,00 | N/A | N/A | 18,00 | 9,00 | N/A | N/A | 54,00 | N/A | N/A | 15,00 | 6,00 | 8,00 |
| 10 | N/A | N/A | N/A | N/A | 0,00 | 2,00 | 2,00 | N/A | N/A | N/A | N/A | N/A | N/A | N/A | 2,00 |  |
| 11 | 2,00 | 0,00 | N/A | 10,00 | 3,00 | 49,00 | 5,00 | 5,00 | N/A | N/A | 57,00 | N/A | N/A | 7,00 | N/A | 7,00 |
| 13 | N/A | N/A | N/A | N/A | N/A | N/A | N/A | 5,00 | N/A | N/A | 29,00 | 14,00 | N/A | 7,00 | N/A |  |
| 14 | N/A | N/A | N/A | N/A | N/A | 23,00 | N/A | N/A | N/A | N/A | N/A | N/A | N/A | N/A | N/A | N/A |
|  | N/A | N/A | N/A | N/A | N/A | 18,00 | N/A | N/A | N/A | N/A | N/A | N/A | N/A | N/A | N/A | N/A |
|  | N/A | N/A | N/A | N/A | N/A | 5,00 | N/A | N/A | N/A | N/A | N/A | N/A | N/A | N/A | N/A | N/A |
| 15 | N/A | N/A | N/A | N/A | N/A | 16,00 | N/A | N/A | 9,00 | N/A | 29,00 | 9,00 | N/A | 4,00 | N/A | 5,00 |
| 16 | 1,00 | 1,00 | N/A | 9,00 | N/A | N/A | 12,00 | 4,00 | 21,00 | N/A | N/A | N/A | N/A | N/A | N/A | N/A |
| 17 | N/A | 0,00 | N/A | N/A | N/A | 28,00 | 6,00 | N/A | 7,00 | N/A | N/A | N/A | N/A | N/A | N/A | N/A |
| 18 | N/A | N/A | N/A | N/A | N/A | 12,00 | N/A | N/A | N/A | N/A | N/A | N/A | N/A | N/A | N/A | N/A |
| 19 | N/A | N/A | N/A | N/A | N/A | 9,00 | 0,00 | N/A | N/A | N/A | 19,00 | N/A | N/A | N/A | N/A | N/A |
| 20 | N/A | N/A | N/A | 3,00 | N/A | N/A | N/A | N/A | N/A | N/A | 101,00 | 17,00 | N/A | 10,00 | N/A | 13,00 |
| 22 | N/A | 0,00 | N/A | 0,00 | 7,00 | 15,00 | N/A | 16,00 | 22,00 | N/A | 24,00 | 16,00 | N/A | 7,00 | N/A | 17,00 |
| 23 | N/A | N/A | N/A | N/A | N/A | N/A | N/A | N/A | N/A | N/A | N/A | N/A | N/A | N/A | N/A | N/A |
| 24 | N/A | N/A | N/A | 2,00 | N/A | 12,00 | 7,00 | N/A | 2,00 | N/A | 40,00 | N/A | N/A | 9,00 | N/A | 1,00 |
| 25 | 3,00 | 0,00 | 25,00 | 2,00 | N/A | N/A | N/A | N/A | N/A | N/A | 43,00 | 11,00 | N/A | 0,00 | 2,00 | 5,00 |
| 28 | N/A | N/A | N/A | 17,00 | N/A | 108,00 | 84,00 | N/A | N/A | N/A | 357,00 | N/A | N/A | N/A | 100,00 | N/A |
| 29 | N/A | 4,00 | 140,00 | 30,00 | N/A | 89,00 | 128,00 | N/A | N/A | N/A | 219,00 | N/A | N/A | N/A | N/A | N/A |
|  | N/A | 0,00 | 30,00 | 2,00 | N/A | 26,00 | 21,00 | N/A | N/A | N/A | 47,00 | N/A | N/A | N/A | N/A | N/A |
| 30 | 1,00 | N/A | N/A | N/A | N/A | 20,00 | N/A | N/A | 5,00 | N/A | N/A | N/A | N/A | N/A | 3,00 | N/A |
| 31 |  | 0,00 | N/A | 2,00 | N/A | 11,00 | 4,00 | N/A | 4,00 | N/A | 23,00 | 8,00 | N/A | N/A | 3,00 | N/A |
| 32 |  |  |  |  |  |  |  |  |  |  |  |  |  |  |  |  |
| 33 |  |  |  |  |  | 0,00 |  |  |  |  |  |  |  | 2,90 |  | 2,03 |
| 34 | N/A | N/A | N/A | N/A | N/A | 28,00 | N/A | N/A | 10,00 | N/A | 19,00 | N/A | N/A | N/A | 5,00 |  |
| 35 | N/A | 0,00 | N/A | N/A | 1,00 | 6,00 | 6,00 | 10,00 | N/A | N/A | 19,00 | 2,00 | N/A | 2,00 | 3,00 | 2,00 |
| 36 | N/A | N/A | N/A | 1,00 | N/A | 34,00 | N/A | N/A | N/A | N/A | 34,00 | N/A | N/A | 3,00 | N/A |  |
| 37 | N/A | 0,00 | N/A | 2,00 | N/A | N/A | N/A | N/A | N/A | N/A | N/A | N/A | N/A | N/A | N/A |  |
| 38 | N/A | N/A | N/A | N/A | N/A | 22,00 | 6,00 | N/A | N/A | N/A | 92,00 | 14,00 | 11,00 | 7,00 | N/A | 2,00 |
|  | N/A | N/A | N/A | N/A | N/A | 1,00 | 2,00 | N/A | N/A | N/A | 12,00 | 4,00 | 2,00 | 2,00 | N/A | 0,00 |
| 39 | 2,00 | N/A | N/A | N/A | 47,00 | N/A | 8,00 | N/A | 14,00 | N/A | 49,00 | 12,00 | N/A | 7,00 | N/A | 3,00 |
| Mean | 2,13 | 0,50 | 58,50 | 6,00 | 19,00 | 24,27 | 18,44 | 14,45 | 13,77 | 4,00 | 63,74 | 12,57 | 6,50 | 7,42 | 15,10 | 7,40 |
| SD | 2,42 | 1,06 | 47,32 | 7,40 | 19,63 | 26,28 | 32,28 | 16,62 | 9,85 | 0,00 | 72,42 | 8,51 | 3,64 | 7,23 | 28,75 | 8,15 |
| ∑ |  |  | 234,00 |  |  |  |  |  |  |  |  |  |  |  |  |  |

**Table 2c:** Data extraction; pre AF= preoperative atrial fibrillation, pre EF= preoperative ejection fraction, av conduit= aortic valve conduit

| **Author** | **renal failure** | **previous cardiac operation** | **pre AF** | **congestive heart failure** | **previous myocardial infarction** | **previous stroke** | **pre creatinine** | **pre EF** | **isolated cerebral perfusion time** | **cerebral perfusion flow** | **ascending and hemiarch** | **elephant trunk** | **aortic root reconstruction** | **aortic root untouched** | **av conduit** | **av replacement** |
| --- | --- | --- | --- | --- | --- | --- | --- | --- | --- | --- | --- | --- | --- | --- | --- | --- |
| 1 | 14,00 | 2,00 | N/A | N/A | N/A | N/A | N/A | N/A | 37,00 | 1,19 | 32,00 | N/A | 22,00 | 9,00 | 8,00 | N/A |
| 3 | N/A | N/A | 32,00 | 102,00 | 24,00 | N/A | 1,10 | 60,00 | N/A | N/A | 14,00 | N/A | 132,00 | N/A | 33,00 | N/A |
|  | N/A | N/A | 22,00 | 47,00 | 5,00 | N/A | 1,10 | 60,00 | N/A | N/A | 1,00 | N/A | 64,00 | N/A | 6,00 | N/A |
| 4 | 0,00 | 2,00 | N/A | N/A | N/A | N/A | N/A | N/A | N/A | N/A | 15,00 | N/A | N/A | N/A | 3,00 | 2,00 |
|  | 2,00 | 3,00 | N/A | N/A | N/A | N/A | N/A | N/A | N/A | N/A | 16,00 | N/A | N/A | N/A | 1,00 | 2,00 |
|  | N/A | N/A | N/A | N/A | N/A | N/A | N/A | N/A | N/A | N/A | 12,00 | N/A | N/A | N/A | 2,00 | 3,00 |
|  | N/A | N/A | N/A | N/A | N/A | N/A | N/A | N/A | N/A | N/A | 19,00 | N/A | N/A | N/A | 2,00 | 1,00 |
| 6 | N/A | N/A | N/A | N/A | N/A | N/A | N/A | N/A | N/A | N/A | 136,00 | N/A | 104,00 | 2,00 | 18,00 |  |
|  | 1,00 | N/A | 1,00 | N/A | N/A | 8,00 | N/A | N/A | N/A | N/A | N/A | N/A | N/A | N/A | N/A | N/A |
|  | 0,00 | N/A | 2,00 | N/A | N/A | 5,00 | N/A | N/A | N/A | N/A | N/A | N/A | N/A | N/A | N/A | N/A |
| 7 | 9,00 | 1,00 | N/A | N/A | N/A | 6,00 | N/A | 61,90 | N/A | N/A | N/A | N/A | 28,00 | N/A | 14,00 | 15,00 |
| 8 | N/A | N/A | N/A | N/A | N/A | 6,00 | 1,40 |  |  |  |  |  |  |  |  |  |
| 10 | N/A | N/A | N/A | N/A | N/A | N/A | N/A | N/A | N/A | N/A | 8,00 | N/A | N/A | N/A | N/A | N/A |
| 11 | N/A | 3,00 | 29,00 | N/A | N/A | N/A | N/A | 54,00 | N/A | N/A | 25,00 | N/A | N/A | N/A | 4,00 | 4,00 |
| 12 | N/A | 1,00 | N/A | N/A | N/A | N/A | N/A | N/A | N/A | N/A | N/A | N/A | N/A | N/A | N/A | N/A |
| 13 | N/A | 1,00 | N/A | N/A | N/A | N/A | N/A | N/A | N/A | N/A | 4,00 | N/A | N/A | N/A | 4,00 | 7,00 |
| 14 | 20,00 | N/A | N/A | N/A | N/A | N/A | N/A | N/A | N/A | N/A | N/A | N/A | 6,00 | N/A | N/A | 2,00 |
|  | 16,00 | N/A | N/A | N/A | N/A | N/A | N/A | N/A | N/A | N/A | N/A | N/A | 5,00 | N/A | N/A | 2,00 |
|  | 4,00 | N/A | N/A | N/A | N/A | N/A | N/A | N/A | N/A | N/A | N/A | N/A | 1,00 | N/A | N/A | N/A |
| 15 | 4,00 | 6,00 | N/A | N/A | N/A | N/A | 1,06 | N/A | 63,00 | N/A | 11,00 | N/A | N/A | N/A | N/A | 6,00 |
| 16 | N/A | 7,00 | N/A | N/A | 2,00 | 16,00 | N/A | 60,00 | 22,00 | N/A | 6,00 | 0,00 | N/A | N/A | 3,00 | 8,00 |
| 17 | 5,00 | 1,00 | N/A | N/A | N/A | N/A | N/A | N/A | N/A | N/A | N/A | N/A | N/A | N/A | N/A | N/A |
|  | N/A | N/A | N/A | N/A | N/A | N/A | N/A | N/A | N/A | N/A | 73,00 | N/A | 0,00 | N/A | N/A | 1,00 |
|  | N/A | N/A | N/A | N/A | N/A | N/A | N/A | N/A | 42,00 | N/A | N/A | N/A | 0,00 | N/A | N/A | 0,00 |
| 18 | 2,00 | N/A | N/A | N/A | 5,00 | N/A | N/A | N/A | N/A | N/A | 4,00 | N/A | 2,00 | 2,00 | N/A | 6,00 |
| 19 | N/A | N/A | N/A | N/A | N/A | 2,00 | N/A | N/A | 57,70 | N/A | 31,00 | N/A | 1,00 | N/A | N/A | 0,00 |
| 20 | 9,00 | N/A | N/A | N/A | N/A | N/A | N/A | N/A | N/A | N/A | 60,00 | N/A | 55,00 | N/A | N/A | N/A |
|  | N/A | N/A | N/A | N/A | N/A | N/A | N/A | N/A | N/A | N/A | 35,00 | N/A | 18,00 | N/A | N/A | 18,00 |
|  | N/A | N/A | N/A | N/A | N/A | N/A | N/A | N/A | N/A | N/A | 25,00 | N/A | 15,00 | N/A | N/A | 15,00 |
| 22 | 7,00 | 4,00 |  | 22,00 | 8,00 | 10,00 | N/A | N/A | 9,00 | N/A | 21,00 | N/A | N/A | N/A | 6,00 | 7,00 |
| 23 | N/A | N/A | N/A | N/A | 2,00 | 1,00 | 2,00 | N/A | N/A | N/A | 53,00 | N/A | N/A | N/A | 0,00 | N/A |
| 24 | 1,00 | N/A | N/A | N/A | N/A | N/A | N/A | N/A | N/A | N/A | N/A | N/A | 2,00 | N/A | N/A | N/A |
| 25 | N/A | N/A | N/A | N/A | N/A | 7,00 | N/A | N/A | 19,00 | N/A | 27,00 | N/A | 5,00 | N/A | N/A | N/A |
| 28 | N/A | 30,00 | N/A | N/A | N/A | N/A | N/A | 49,00 | N/A | N/A | N/A | N/A | 327,00 | N/A | N/A | 299,00 |
| 29 | N/A | N/A | N/A | N/A | N/A | N/A | N/A | N/A | 289,00 | N/A | 199,00 | N/A | N/A | N/A | N/A | 28,00 |
|  | N/A | N/A | N/A | N/A | N/A | N/A | N/A | N/A | 55,00 | N/A | 41,00 |  |  |  |  | 10,00 |
| 30 | 14,00 | 2,00 | N/A | N/A | 1,00 | N/A | N/A | N/A |  |  | 23,00 |  | 0,00 |  |  | 0,00 |
| 31 | N/A |  |  |  | 6,00 | 2,00 |  |  |  |  |  |  |  |  |  |  |
| 33 | 2,03 | N/A | 6,96 | 2,90 | 4,93 | 2,03 | N/A | N/A | 4,06 | N/A | 18,85 | N/A | N/A | N/A | N/A | 4,35 |
| 35 | 1,00 | N/A | N/A | N/A | N/A | 2,00 | N/A | 40,00 | N/A | N/A | 19,00 | N/A | N/A | N/A | 1,00 | 2,00 |
| 36 | 17,00 | N/A | N/A | N/A | N/A | N/A | N/A | N/A | N/A | N/A | 44,00 | N/A | 1,00 | N/A | N/A | 3,00 |
| 37 | N/A | 0,00 | N/A | N/A | N/A | N/A | N/A | N/A | 65,00 | N/A | 23,00 | N/A | 0,00 | N/A | N/A | N/A |
| 38 | 3,00 | N/A | 2,00 | N/A | 5,00 | N/A | N/A | N/A | N/A | N/A | 45,00 | N/A | 101,00 | 2,00 | 14,00 | N/A |
|  | 0,00 | N/A | 1,00 | N/A | 0,00 | N/A | N/A | N/A | N/A | N/A | 7,00 | N/A | 14,00 | 0,00 | 4,00 | N/A |
| 39 | 2,00 | N/A | N/A | N/A | 9,00 | 8,00 | N/A | 62,90 | N/A | N/A | N/A | 62,00 | N/A | N/A | 6,00 | 1,00 |
| Mean | 6,05 | 4,50 | 12,00 | 43,48 | 5,99 | 5,77 | 1,33 | 55,98 | 60,25 | N/A | N/A | 31,00 | 39,26 | 3,00 | 7,17 | 17,17 |
| SD | 6,14 | 7,33 | 12,53 | 37,23 | 6,01 | 4,06 | 0,36 | 7,41 | 75,20 | N/A | N/A | 31,00 | 71,97 | 3,10 | 7,92 | 56,74 |

**Table 2d:** Data extraction; cabg= coronary artery bypass graft, icu= intensive care unit, cpb= cardiopulmonary bypass

| **Author** | **cabg** | **circulatory arrest** | **icu stay** | **ventilation time** | **reintubation** | **prolonged ventilation** | **chest tube drainage** | **reexploration for bleeding** | **length of stay** | **op duration** | **cpb** | **femoral cannulation** | **axillary cannulation** | **aorta cannulation** | **circulatory arrest time** | **crossclamp time** |
| --- | --- | --- | --- | --- | --- | --- | --- | --- | --- | --- | --- | --- | --- | --- | --- | --- |
| 1 | 2,00 | N/A | 5,00 | 46,00 | N/A | N/A | 760,00 | 5,00 | N/A | N/A | N/A | N/A | N/A | N/A | 37,00 | 103,00 |
| 2 | N/A | N/A | N/A | N/A | N/A | N/A | N/A | N/A | N/A | N/A | N/A | N/A | N/A | N/A | 37,00 | 83,00 |
| 3 | N/A | N/A | N/A | N/A | N/A | 74,00 | N/A | 10,00 | 12,00 | N/A | N/A | N/A | N/A | N/A | 35,00 | 143,00 |
|  | N/A | N/A | N/A | N/A | N/A | 35,00 | N/A | 6,00 | 13,00 | N/A | N/A | N/A | N/A | N/A | 31,00 | 141,00 |
| 4 | 6,00 | N/A | N/A | N/A | N/A | N/A | N/A | N/A | N/A | N/A | N/A | N/A | N/A | N/A | 32,80 | 90,00 |
|  | 3,00 | N/A | N/A | N/A | N/A | N/A | N/A | N/A | N/A | N/A | N/A | N/A | N/A | N/A | 33,80 | 86,70 |
|  | 4,00 | 20,00 | N/A | N/A | N/A | N/A | N/A | N/A | N/A | N/A | N/A | N/A | N/A | N/A | 36,00 | 93,00 |
|  | 5,00 | 27,00 | N/A | N/A | N/A | N/A | N/A | N/A | N/A | N/A | N/A | N/A | N/A | N/A | 31,00 | 86,00 |
|  | N/A | N/A | 1,00 | N/A | N/A | N/A | N/A | N/A | N/A | N/A | N/A | N/A | N/A | N/A |  |  |
| 6 | N/A | N/A | 9,20 | N/A | N/A | 51,40 | N/A | 6,00 | 19,70 | N/A | N/A | N/A | N/A | N/A | 28,20 | 158,10 |
|  | N/A | N/A | 7,80 | N/A | 4,00 | 51,50 | N/A | 2,00 | 19,60 | 7,20 | N/A | N/A | N/A | N/A | 26,50 | 147,60 |
|  | N/A | N/A | 9,00 | N/A | 7,00 | 51,20 | N/A | 4,00 | 19,90 | 8,40 | N/A | N/A | N/A | N/A | 30,90 | 175,90 |
| 7 | 2,00 | N/A | 87,80 | 20,30 | N/A | N/A | 514,70 | 1,00 | 19,60 | N/A | N/A | N/A | N/A | N/A | 3,70 | 47,90 |
| 8 | N/A | N/A | N/A | N/A | N/A | 36,00 | N/A | N/A | N/A | N/A | N/A | N/A | N/A | N/A | 25,30 | 90,00 |
| 9 | N/A | 15,40 | N/A | 13,00 | N/A | N/A | N/A | N/A | 12,20 | 143,40 | N/A | N/A | N/A | N/A | 18,70 | N/A |
| 10 | N/A | 15,20 | N/A | N/A | N/A | 2,00 | N/A | N/A | 39,00 | 280,00 | 155,00 | N/A | N/A | N/A | 31,00 | 104,00 |
| 11 | 5,00 | 5,00 | 6,00 | N/A | N/A | 46,00 | N/A | 13,00 | 12,00 | N/A | 156,00 | N/A | N/A | N/A | 38,00 | 83,00 |
| 12 | N/A | N/A | N/A | N/A | N/A | N/A | N/A | N/A | N/A | N/A | N/A | N/A | N/A | N/A | 50,60 | N/A |
| 13 | N/A | 22,60 | N/A | N/A | N/A | N/A | N/A | N/A | 15,90 | N/A | 154,30 | 16,00 | 11,00 | 4,00 | 22,60 | 77,60 |
| 14 | 4,00 | 99,70 | N/A | N/A | N/A | N/A | 3712,00 | N/A | 41,20 | 537,20 | 291,60 | 3,00 | N/A | N/A | 99,70 | N/A |
|  | 3,00 | 99,00 | N/A | N/A | N/A | N/A | 3394,00 | N/A | 37,00 | 509,10 | 279,70 | 3,00 | 1,00 | N/A | 99,00 | N/A |
|  | 1,00 | 104,00 | N/A | N/A | N/A | N/A | 5705,00 | N/A | 68,40 | 720,10 | 368,50 | N/A | 1,00 | N/A | 194,00 | N/A |
|  | N/A | N/A | N/A | N/A | N/A | N/A | N/A | N/A | N/A | 662,60 | 325,00 | N/A | N/A | N/A | 210,30 | N/A |
|  | N/A | N/A | N/A | N/A | N/A | N/A | N/A | N/A | N/A | 494,50 | 280,30 | N/A | N/A | N/A | 171,40 | N/A |
| 15 | 2,00 | N/A | 4,45 | N/A | N/A | N/A | N/A | N/A | 26,30 | 368,00 | 200,00 | 1,00 | N/A | N/A | 53,00 | 131,00 |
| 16 | 7,00 | N/A | 5,00 | 87,00 | 8,00 | N/A | 850,00 | 9,00 | N/A | 253,00 | 155,00 | 11,00 | N/A | 5,00 | 29,00 | 79,00 |
| 17 | N/A | N/A | N/A | N/A | N/A | N/A | N/A | N/A | N/A | N/A | N/A | N/A | N/A | N/A | 29,00 | N/A |
|  | 1,00 | N/A | N/A | N/A | N/A | N/A | N/A | N/A | N/A | 294,00 | N/A | 69,00 | 0,00 | 1,00 | N/A | N/A |
|  | 0,00 | N/A | N/A | N/A | N/A | N/A | N/A | N/A | N/A | 488,00 | N/A | 0,00 | 4,00 | 0,00 | N/A | N/A |
| 18 | 1,00 | N/A | 8,00 | N/A | N/A | 12,00 | N/A | 7,00 | 20,00 | N/A | 153,00 | 38,00 | 4,00 | N/A | 28,00 | 87,00 |
| 19 | 2,00 | N/A | 8,00 | N/A | N/A | 9,00 | 1489,70 | N/A | 25,30 | 347,10 | 206,80 | N/A | N/A | N/A | 58,00 | N/A |
| 20 | 9,00 | N/A | 10,70 | 99,90 | N/A | N/A | N/A | N/A | N/A | 309,20 | 191,90 | N/A | N/A | N/A | 29,70 | 96,60 |
|  | 6,00 | N/A | 11,40 | 123,30 | N/A | N/A | N/A | N/A | N/A | 340,00 | 202,50 | N/A | N/A | N/A | 25,90 | 101,60 |
|  | 3,00 | N/A | 10,10 | 75,00 | N/A | N/A | N/A | N/A | N/A | 276,10 | 180,10 | N/A | N/A | N/A | 33,70 | 91,00 |
| 21 | N/A | N/A | N/A | N/A | N/A | N/A | N/A | N/A | N/A | N/A | N/A | N/A | N/A | N/A | 31,00 | N/A |
|  | N/A | N/A | N/A | N/A | N/A | N/A | N/A | N/A | N/A | N/A | N/A | N/A | N/A | N/A | 30,50 | N/A |
| 22 | 2,00 | 71,00 | N/A | N/A | N/A | 8,00 | N/A | 6,00 | N/A | N/A | N/A | N/A | N/A | N/A | 32,00 | 71,00 |
| 23 | 4,00 | 101,20 | 7,00 | N/A | N/A | 22,00 | N/A | N/A | N/A | 320,60 | 168,50 | N/A | N/A | N/A | 101,20 | N/A |
| 24 |  | 96,00 | 2,50 | N/A | 6,00 | 14,00 | N/A | 4,00 | N/A | 258,00 | 139,00 | N/A | N/A | N/A | N/A | 96,00 |
| 25 | 6,00 | 44,00 | N/A | N/A | N/A | N/A | N/A | N/A | N/A | N/A | 182,00 | N/A | N/A | N/A | 44,00 | 87,00 |
| 26 | N/A | N/A | N/A | N/A | N/A | N/A | N/A | N/A | N/A | N/A | N/A | N/A | N/A | N/A | 46,00 | N/A |
| 27 | N/A | N/A | N/A | N/A | N/A | N/A | N/A | N/A | N/A | N/A | N/A | N/A | N/A | N/A | 51,00 | 93,00 |
| 28 | 37,00 | 33,00 | 7,00 | 96,00 | N/A | N/A | N/A | N/A | N/A | N/A | 215,00 | 187,00 | 176,00 | N/A | 33,00 | 98,00 |
| 29 | 69,00 | 29,70 | 18,00 | N/A | N/A | 189,00 | N/A | 78,00 | 18,00 | 322,10 | N/A | N/A | N/A | N/A | 29,70 | N/A |
|  | 8,00 | 29,00 | 12,50 | N/A | N/A | 35,00 | N/A | 19,00 | 17,70 | 293,30 | N/A | N/A | N/A | N/A | 29,00 | N/A |
| 30 | 1,00 | 112,00 | N/A | N/A | N/A | N/A | N/A | 3,00 | 32,20 | N/A | 185,00 | N/A | N/A | N/A | 29,50 | 92,00 |
| 32 | N/A | N/A | N/A | N/A | N/A | N/A | N/A | N/A | N/A | N/A | N/A | N/A | N/A | N/A | 27,50 | 89,50 |
| 33 | 8,12 | 13,00 | N/A | N/A | N/A | 6,96 | N/A | 4,06 | 14,00 | N/A | 148,00 | 24,94 | 2,32 | 0,87 | 13,00 | 99,00 |
| 34 |  | 30,00 | N/A | N/A | N/A | N/A | 475,00 | 3,00 | N/A | 238,00 | 150,00 | N/A | N/A | N/A | 30,00 | 70,00 |
| 35 | 4,00 | 16,00 | N/A | N/A | N/A | N/A | N/A | 2,00 | N/A | N/A | 177,00 | N/A | N/A | N/A | 16,00 | N/A |
| 36 | 2,00 | N/A | 8,00 | N/A | N/A | N/A | N/A | 4,00 | 28,50 | 264,00 | 132,50 | N/A | N/A | N/A | N/A | 88,50 |
| 37 | N/A | N/A | 7,00 | N/A | N/A | N/A | N/A | 0,00 | 38,00 | 404,00 | 193,00 | N/A | N/A | N/A | 71,00 | 135,00 |
| 38 | N/A | N/A | 6,00 | 49,30 | 11,00 | N/A | N/A | 6,00 | 20,50 | 462,00 | 228,30 | 33,00 | 30,00 | 2,00 | 28,00 | 157,80 |
|  | N/A | N/A | 5,00 | 67,60 | 0,00 | N/A | N/A | 0,00 | 14,40 | 426,00 | 219,30 | 8,00 | 5,00 | 0,00 | 24,80 | 159,70 |
| 39 | 5,00 | N/A | 5,00 | N/A | N/A | 19,00 | N/A | 1,00 | 13,00 | N/A | 178,20 | 63,00 | N/A | 1,00 | 20,00 | 102,50 |
| 41 | N/A | N/A | N/A | N/A | N/A | N/A | N/A | N/A | N/A | N/A | N/A | N/A | N/A | N/A | 18,00 | 98,00 |
| Mean | 7,07 | 49,14 | 10,89 | 67,74 | 6,00 | 38,94 | 2112,55 | 8,39 | 23,90 | 347,15 | 200,55 | 35,15 | 23,43 | 1,73 | 45,39 | 103,59 |
| SD | 13,16 | 37,09 | 16,40 | 33,77 | 3,42 | 42,41 | 1808,04 | 15,44 | 12,74 | 163,04 | 57,78 | 49,03 | 51,55 | 1,72 | 41,61 | 28,94 |
| ∑ | 212,12 |  |  |  |  |  |  |  |  |  |  | 456,94 | 234,32 | 13,87 |  |  |

**Table 2e:** Data extraction; af= atrial fibrillation

| **mean core temperature** | **total arch replacement** | **aortic root replacement** | **no post complications** | **infectious** | **sepsis** | **delirium** | **cerebral** | **bleeding** | **af** | **respiratory failure** | **renal failure requiring hemofiltration** | **tracheotomy** | **transient neurologic deficit** | **permanent neurologic deficit** | **postoperative stroke** |
| --- | --- | --- | --- | --- | --- | --- | --- | --- | --- | --- | --- | --- | --- | --- | --- |
| 28,00 | 7,00 | 8,00 | N/A | N/A | N/A | N/A | N/A | N/A | N/A | N/A | 5,00 | N/A | 3,00 | 2,00 | 2,00 |
| 24,10 | N/A | 4,00 | N/A | N/A | N/A | N/A | N/A | N/A | N/A | N/A | N/A | N/A | N/A | N/A | 9,00 |
| N/A | 14,00 | 33,00 | N/A | N/A | N/A | N/A | N/A | N/A | N/A | N/A | 31,00 | N/A | N/A | N/A | 23,00 |
| N/A | 1,00 | 6,00 | N/A | N/A | N/A | N/A | N/A | N/A | N/A | N/A | 11,00 | N/A | N/A | N/A | 9,00 |
| 18,00 | 4,00 | 3,00 | N/A | N/A | N/A | N/A | N/A | N/A | N/A | N/A | N/A | N/A | N/A | N/A | 19,00 |
| N/A | N/A | 1,00 | N/A | N/A | N/A | N/A | N/A | N/A | N/A | N/A | N/A | N/A | N/A | N/A | N/A |
| N/A | N/A | N/A | N/A | N/A | N/A | N/A | N/A | N/A | N/A | N/A | N/A | N/A | N/A | N/A | N/A |
| N/A | N/A | N/A | N/A | N/A | N/A | N/A | N/A | N/A | N/A | N/A | N/A | N/A | N/A | N/A | N/A |
| N/A | N/A | N/A | N/A | 10,00 | N/A | N/A | N/A | 2,00 | N/A | N/A | 14,00 | N/A | N/A | N/A | 10,00 |
| N/A | N/A | N/A | N/A | 3,00 | N/A | N/A | N/A | 4,00 | N/A | N/A | 8,00 | N/A | N/A | N/A | 3,00 |
| 25,00 | 0,00 | 0,00 | 7,00 | N/A | N/A | 1,00 | 2,00 | 2,00 | 4,00 | N/A | N/A | N/A | N/A | N/A | 1,00 |
| N/A | 34,00 | 19,00 | N/A | 2,00 | N/A | N/A | N/A | N/A | N/A | N/A | 37,00 | 10,00 | N/A | 1,00 | 7,00 |
| N/A | N/A | N/A | N/A | 1,00 | N/A | N/A | N/A | N/A | N/A | N/A | 20,00 | N/A | N/A | 0,00 | 7,00 |
| N/A | N/A | N/A | N/A | 1,00 | N/A | N/A | N/A | N/A | N/A | N/A | 17,00 | N/A | N/A | 1,00 | 1,00 |
| 26,50 | 67,00 | 16,00 | N/A | 1,00 | 7,00 | N/A | N/A | N/A | N/A | 47,00 | 25,00 | 7,00 | 2,00 | 2,00 | 2,00 |
| N/A | 8,00 | 6,00 | N/A | N/A | 5,00 | N/A | N/A | N/A | N/A | N/A | 20,00 | 11,00 | N/A | N/A | 11,00 |
| 28,00 | 0,00 | N/A | N/A | N/A | N/A | N/A | N/A | N/A | N/A | N/A | N/A | N/A | 1,00 | 1,00 | 0,00 |
| 25,40 | 0,00 | N/A | N/A | N/A | N/A | N/A | N/A | N/A | N/A | N/A | N/A | 1,00 | N/A | N/A | 1,00 |
| 19,50 | N/A | 4,00 | N/A | 1,00 | N/A | N/A | N/A | N/A | 28,00 | N/A | 13,00 | N/A | 5,00 | N/A | 7,00 |
| N/A | 27,00 | 8,00 | N/A | N/A | N/A | N/A | N/A | N/A | N/A | N/A | N/A | N/A | N/A | N/A | 45,00 |
| N/A | N/A | 4,00 | N/A | N/A | N/A | N/A | N/A | N/A | N/A | N/A | 1,00 | N/A | N/A | N/A | 6,00 |
| N/A | 8,00 | 6,00 | N/A | N/A | N/A | N/A | N/A | 2,00 | N/A | N/A | 6,00 | 3,00 | 6,00 | 9,00 | 9,00 |
| N/A | N/A | N/A | N/A | N/A | N/A | N/A | N/A | 1,00 | N/A | N/A | 4,00 | 2,00 | 6,00 | 7,00 | N/A |
| N/A | N/A | N/A | N/A | N/A | N/A | N/A | N/A | 1,00 | N/A | N/A | 2,00 | 1,00 | 0,00 | 2,00 | N/A |
| 23,10 | N/A | N/A | N/A | N/A | N/A | N/A | N/A | N/A | N/A | N/A | N/A | N/A | N/A | N/A | N/A |
| 23,70 | N/A | N/A | N/A | N/A | N/A | N/A | N/A | N/A | N/A | N/A | N/A | N/A | N/A | N/A | N/A |
| 25,00 | 11,00 | N/A | N/A | 3,00 | N/A | N/A | N/A | N/A | N/A | 1,00 | N/A | N/A | N/A | N/A | 4,00 |
| 22,00 | 6,00 | 3,00 | N/A | 7,00 | 5,00 | 8,00 | N/A | N/A | 8,00 | N/A | 18,00 | 14,00 | N/A | N/A | 8,00 |
| 20,00 | 4,00 | 0,00 | N/A | N/A | N/A | N/A | N/A | N/A | N/A | N/A | N/A | N/A | N/A | N/A | 7,00 |
| N/A | N/A | N/A | N/A | N/A | N/A | N/A | N/A | N/A | N/A | N/A | N/A | N/A | N/A | N/A | N/A |
| N/A | N/A | N/A | N/A | N/A | N/A | N/A | N/A | N/A | N/A | N/A | N/A | N/A | N/A | N/A | N/A |
| 19,00 | 1,00 | 3,00 | N/A | 1,00 | 5,00 | N/A | 3,00 | N/A | N/A | N/A | 8,00 | 5,00 | 10,00 | 1,00 | 10,00 |
| 24,00 | 0,00 | 1,00 | N/A | N/A | N/A | N/A | N/A | N/A | N/A | N/A | N/A | N/A | 1,00 | N/A | 8,00 |
| 26,40 | 11,00 | 33,00 | N/A | 43,00 | N/A | N/A | N/A | N/A | N/A | N/A | 44,00 | N/A | 5,00 | N/A | 23,00 |
| 26,40 | N/A | N/A | N/A | 27,00 | N/A | N/A | N/A | N/A | N/A | N/A | 28,00 | N/A | N/A | N/A | N/A |
| 26,30 | N/A | N/A | N/A | 16,00 | N/A | N/A | N/A | N/A | N/A | N/A | 16,00 | N/A | N/A | N/A | N/A |
| 20,40 | 10,00 | 1,00 | N/A | N/A | N/A | N/A | N/A | N/A | N/A | N/A | 3,00 | N/A | N/A | N/A | 8,00 |
| 21,50 | 1,00 | 0,00 | N/A | N/A | N/A | N/A | N/A | N/A | N/A | N/A | N/A | N/A | N/A | N/A | 5,00 |
| 27,00 | 3,00 | 8,00 | 0,00 | 24,00 | 6,00 | N/A | 10,00 | 12,00 | N/A | N/A | 9,00 | N/A | N/A | N/A | 6,00 |
| 29,00 | 9,00 | 0,00 | N/A | N/A | N/A | N/A | 3,00 | N/A | N/A | N/A | N/A | N/A | N/A | N/A | 3,00 |
| N/A | 18,00 | 2,00 | N/A | 5,00 | N/A | 22,00 | N/A | 4,00 | N/A | N/A | 2,00 | N/A | 1,00 | 0,00 | 0,00 |
| 19,70 | 6,00 | 8,00 | N/A | N/A | 8,00 | N/A | N/A | 2,00 | N/A | 4,00 | 14,00 | N/A | N/A | N/A | 7,00 |
| 19,00 | 5,00 | 2,00 | N/A | N/A | N/A | N/A | N/A | N/A | N/A | N/A | N/A | N/A | N/A | N/A | 2,00 |
| N/A | 6,00 | 8,00 | N/A | N/A | N/A | N/A | N/A | N/A | N/A | N/A | N/A | N/A | N/A | N/A | 3,00 |
| 24,00 | 31,00 | 73,00 | N/A | N/A | N/A | 129,00 | N/A | 88,00 | N/A | N/A | N/A | N/A | N/A | N/A | 73,00 |
| 24,40 | 54,00 | 17,00 | N/A | N/A | N/A | N/A | N/A | N/A | N/A | N/A | N/A | N/A | N/A | N/A | N/A |
| 24,10 | 8,00 | 2,00 | N/A | N/A | N/A | N/A | N/A | N/A | N/A | N/A | N/A | N/A | N/A | N/A | N/A |
| 18,00 | 3,00 | 5,00 | N/A | N/A | N/A | N/A | N/A | N/A | N/A | N/A | N/A | N/A | N/A | N/A | 8,00 |
| 19,00 | 1,00 | 0,00 | N/A | 4,00 | N/A | N/A | N/A | N/A | N/A | N/A | 3,00 | 1,00 | N/A | N/A | 2,00 |
| 23,00 | 6,00 | N/A | N/A | N/A | N/A | N/A | N/A | N/A | N/A | N/A | N/A | N/A | N/A | N/A | 9,00 |
| N/A | 1,00 | 2,00 | N/A | 2,00 | 0,58 | N/A | N/A | N/A | 8,99 | N/A | 4,93 | N/A | N/A | N/A | 6,00 |
| 25,00 | 0,00 | 0,00 | N/A | N/A | N/A | N/A | N/A | N/A | N/A | 5,00 | 1,00 | N/A | N/A | 2,00 | 2,00 |
| 21,00 | 0,00 | 1,00 | N/A | 0,00 | 1,00 | N/A | N/A | 2,00 | N/A | 7,00 | 0,00 | 3,00 | N/A | 0,00 | 1,00 |
| N/A | 7,00 | 1,00 | N/A | 2,00 | N/A | N/A | N/A | N/A | N/A | 7,00 | 2,00 | N/A | N/A | N/A | 3,00 |
| 25,00 | 1,00 | 0,00 | N/A | 0,00 | N/A | N/A | N/A | N/A | N/A | 0,00 | 0,00 | N/A | N/A | N/A | 0,00 |
| N/A | 31,00 | 15,00 | N/A | 2,00 | N/A | N/A | N/A | N/A | N/A | N/A | 31,00 | 10,00 | N/A | 1,00 | 6,00 |
| N/A | 3,00 | 4,00 | N/A | 0,00 | N/A | N/A | N/A | N/A | N/A | N/A | 6,00 | 0,00 | N/A | 0,00 | 1,00 |
| 26,40 | 65,00 | 9,00 | N/A | 3,00 | N/A | N/A | N/A | N/A | N/A | 5,00 | 17,00 | N/A | N/A | N/A | 6,00 |
| 21,30 | 3,00 | 1,00 | N/A | N/A | N/A | N/A | N/A | N/A | N/A | N/A | N/A | N/A | N/A | N/A | 1,00 |
| 17,00 | 8,00 | 11,00 | N/A | N/A | N/A | N/A | N/A | N/A | N/A | N/A | N/A | N/A | N/A | N/A | 23,00 |
| 23,20 | 11,50 | 7,81 | 3,50 | 6,87 | 4,70 | 40,00 | 4,50 | 10,91 | 12,25 | 9,50 | 12,76 | 5,23 | 3,64 | 1,93 | 8,66 |
| 3,21 | 16,49 | 12,77 | 3,50 | 10,56 | 2,47 | 51,94 | 3,20 | 24,56 | 9,28 | 14,37 | 11,40 | 4,47 | 2,90 | 2,52 | 12,38 |
| N/A | 483,00 | 328,00 | 7,00 | 158,00 | 37,58 | 160,00 | N/A | N/A | N/A | N/A | N/A | N/A | N/A | N/A | N/A |

**Table 2f**: Data extraction;gi bleeding= gastrointestinal bleeding, los= length of stay, ic hemorrhage= intracranial hemorrhage, los= low output syndrome

| **gi bleeding** | **limb ischemia** | **myocardial infarction** | **los** | **readmission** | **30d mortality** | **in hospital death** | **late survival** | **multiorgan failure** | **respiratory failure** | **sepsis** | **ic hemorrhage** | **gi bleeding** | **los** | **aortic rupture** |
| --- | --- | --- | --- | --- | --- | --- | --- | --- | --- | --- | --- | --- | --- | --- |
| N/A | N/A | N/A | N/A | N/A | 10,00 | N/A | 24,18 | 5,00 | 2,00 | N/A | 1,00 | 1,00 | 1,00 | N/A |
| N/A | N/A | N/A | N/A | N/A | 12,00 | N/A | N/A | N/A | N/A | N/A | N/A | N/A | N/A | N/A |
| N/A | N/A | N/A | N/A | N/A | 35,00 | N/A | 117,15 | N/A | N/A | N/A | N/A | N/A | N/A | N/A |
| N/A | N/A | N/A | N/A | N/A | 20,00 | N/A | 42,70 | N/A | N/A | N/A | N/A | N/A | N/A | N/A |
| N/A | N/A | N/A | N/A | N/A | N/A | 21,00 | 16,40 | N/A | N/A | N/A | N/A | N/A | N/A | N/A |
| N/A | N/A | N/A | N/A | N/A | N/A | 10,00 | 25,20 | N/A | N/A | N/A | N/A | N/A | N/A | N/A |
| N/A | N/A | N/A | N/A | N/A | N/A | N/A | N/A | N/A | N/A | N/A | N/A | N/A | N/A | N/A |
| N/A | N/A | N/A | N/A | N/A | N/A | N/A | N/A | N/A | N/A | N/A | N/A | N/A | N/A | N/A |
| N/A | N/A | N/A | N/A | N/A | N/A | N/A | 3,50 | N/A | N/A | N/A | N/A | N/A | N/A | N/A |
| N/A | N/A | N/A | N/A | N/A | N/A | N/A | 7,80 | N/A | N/A | N/A | N/A | N/A | N/A | N/A |
| N/A | N/A | N/A | N/A | N/A | 2,00 | N/A | 12,96 | N/A | N/A | N/A | N/A | N/A | N/A | N/A |
| 4,00 | 0,00 | N/A | N/A | N/A | 28,00 | N/A | N/A | N/A | N/A | N/A | N/A | N/A | N/A | N/A |
| 2,00 | N/A | N/A | N/A | N/A | 15,00 | N/A | N/A | N/A | N/A | N/A | N/A | N/A | N/A | N/A |
| 2,00 | N/A | N/A | N/A | N/A | 13,00 | N/A | N/A | N/A | N/A | N/A | N/A | N/A | N/A | N/A |
| 2,00 | N/A | 1,00 | N/A | N/A | 5,00 | N/A | N/A | N/A | N/A | N/A | N/A | N/A | N/A | N/A |
| N/A | N/A | N/A | 12,00 | N/A | 26,00 | N/A | 32,80 | 0,00 | N/A | N/A | N/A | N/A | 12,00 | 4,00 |
| N/A | N/A | N/A | N/A | N/A | N/A | 1,00 | 25,90 | N/A | N/A | N/A | N/A | N/A | N/A | N/A |
| N/A | N/A | N/A | N/A | N/A | 0,00 | N/A | 9,00 | N/A | N/A | N/A | N/A | N/A | N/A | N/A |
| 4,00 | 2,00 | 0,00 | N/A | N/A | 17,00 | N/A | 56,16 | 3,00 | N/A | 1,00 | 7,00 | N/A | 3,00 | N/A |
| N/A | N/A | N/A | N/A | N/A | 53,00 | N/A | N/A | N/A | N/A | N/A | N/A | N/A | N/A | N/A |
| N/A | N/A | N/A | N/A | N/A | 5,00 | N/A | 23,25 | N/A | N/A | N/A | N/A | N/A | N/A | N/A |
| N/A | N/A | N/A | N/A | N/A | N/A | 4,00 | N/A | N/A | 2,00 | 1,00 | 3,00 | N/A | N/A | 2,00 |
| N/A | N/A | N/A | N/A | N/A | N/A | 3,00 | N/A | N/A | 2,00 | 1,00 | 2,00 | N/A | N/A | 2,00 |
| N/A | N/A | N/A | N/A | N/A | N/A | 1,00 | N/A | N/A | 0,00 | 0,00 | 1,00 | N/A | N/A | 0,00 |
| N/A | N/A | N/A | N/A | N/A | N/A | N/A | N/A | N/A | N/A | N/A | N/A | N/A | N/A | N/A |
| N/A | N/A | N/A | N/A | N/A | N/A | N/A | N/A | N/A | N/A | N/A | N/A | N/A | N/A | N/A |
| 3,00 | N/A | 1,00 | N/A | N/A | 6,00 | N/A | 36,96 | N/A | N/A | N/A | N/A | N/A | N/A | N/A |
| N/A | N/A | N/A | N/A | 7,00 | 19,00 | N/A | N/A | 5,00 | N/A | 1,00 | 1,00 | N/A | N/A | N/A |
| N/A | N/A | N/A | N/A | N/A | 5,00 | N/A | 63,14 | N/A | 6,00 | N/A | 1,00 | N/A | N/A | N/A |
| N/A | N/A | N/A | N/A | N/A | N/A | N/A | N/A | N/A | N/A | N/A | N/A | N/A | N/A | N/A |
| N/A | N/A | N/A | N/A | N/A | N/A | N/A | N/A | N/A | N/A | N/A | N/A | N/A | N/A | N/A |
| N/A | N/A | N/A | N/A | N/A | N/A | 7,00 | 36,90 | 1,00 | N/A | 2,00 | N/A | 1,00 | N/A | N/A |
| N/A | N/A | N/A | N/A | N/A | N/A | 5,00 | N/A | N/A | N/A | N/A | N/A | N/A | N/A | N/A |
| N/A | N/A | 6,00 | N/A | N/A | N/A | 26,00 | N/A | N/A | N/A | N/A | N/A | N/A | N/A | N/A |
| N/A | N/A | 2,00 | N/A | N/A | N/A | 13,00 | N/A | N/A | N/A | N/A | N/A | N/A | N/A | N/A |
| N/A | N/A | 4,00 | N/A | N/A | N/A | 13,00 | N/A | N/A | N/A | N/A | N/A | N/A | N/A | N/A |
| N/A | N/A | N/A | N/A | N/A | 5,00 | N/A | 53,10 | N/A | N/A | N/A | N/A | N/A | N/A | N/A |
| N/A | N/A | N/A | N/A | N/A | 3,00 | N/A | 26,56 | N/A | N/A | N/A | N/A | N/A | N/A | N/A |
| 0,00 | N/A | N/A | N/A | N/A | 20,00 | N/A | N/A | N/A | N/A | N/A | N/A | N/A | N/A | N/A |
| N/A | N/A | N/A | N/A | N/A | 7,00 | N/A | N/A | N/A | N/A | N/A | N/A | N/A | N/A | N/A |
| N/A | 0,00 | N/A | N/A | N/A | 2,00 | N/A | N/A | N/A | N/A | N/A | N/A | N/A | N/A | N/A |
| N/A | 1,00 | N/A | 3,00 | N/A | 23,00 | N/A | 29,07 | 5,00 | 3,00 | 1,00 | 2,00 | N/A | 3,00 | 2,00 |
| N/A | N/A | N/A | N/A | N/A | 14,00 | N/A | 22,23 | N/A | N/A | N/A | N/A | N/A | N/A | N/A |
| N/A | N/A | N/A | N/A | N/A | N/A | 35,00 | 12,19 | N/A | N/A | N/A | N/A | N/A | N/A | N/A |
| N/A | N/A | N/A | N/A | N/A | 103,00 | N/A | 264,00 | N/A | N/A | N/A | N/A | N/A | N/A | N/A |
| N/A | N/A | N/A | N/A | N/A | 60,00 | N/A | N/A | N/A | N/A | N/A | N/A | N/A | N/A | N/A |
| N/A | N/A | N/A | N/A | N/A | 29,00 | N/A | N/A | N/A | N/A | N/A | N/A | N/A | N/A | N/A |
| N/A | N/A | N/A | N/A | N/A | 12,00 | N/A | 26,00 | N/A | N/A | N/A | N/A | N/A | N/A | N/A |
| N/A | N/A | N/A | N/A | N/A | N/A | 3,00 | 15,84 | N/A | N/A | N/A | N/A | N/A | N/A | N/A |
| N/A | N/A | N/A | N/A | N/A | N/A | 7,00 | N/A | N/A | N/A | N/A | N/A | N/A | N/A | N/A |
| N/A | 0,00 | 0,87 | N/A | N/A | 7,00 | N/A | 17,98 | N/A | 0,00 | 4,06 | 0,00 | N/A | 20,88 | N/A |
| N/A | N/A | N/A | N/A | N/A | 2,00 | N/A | 20,00 | N/A | N/A | N/A | N/A | N/A | N/A | N/A |
| 0,00 | N/A | 0,00 | N/A | N/A | 0,00 | N/A | 21,00 | N/A | N/A | N/A | N/A | N/A | N/A | N/A |
| 1,00 | N/A | N/A | N/A | N/A | 2,00 | N/A | 45,76 | N/A | 4,00 | N/A | 1,00 | N/A | 1,00 | 2,00 |
| N/A | N/A | N/A | N/A | N/A | 0,00 | N/A | 22,56 | N/A | N/A | N/A | N/A | N/A | N/A | N/A |
| 1,00 | 0,00 | N/A | N/A | N/A | 22,00 | N/A | N/A | N/A | N/A | N/A | N/A | N/A | N/A | N/A |
| 0,00 | 0,00 | N/A | N/A | N/A | 6,00 | N/A | N/A | N/A | N/A | N/A | N/A | N/A | N/A | N/A |
| N/A | N/A | N/A | 6,00 | N/A | N/A | 7,00 | N/A | N/A | N/A | N/A | N/A | N/A | N/A | N/A |
| N/A | N/A | N/A | N/A | N/A | 0,00 | N/A | 10,01 | N/A | N/A | N/A | N/A | N/A | N/A | N/A |
| N/A | N/A | N/A | N/A | N/A | N/A | 25,00 | 69,69 | N/A | N/A | N/A | N/A | N/A | N/A | N/A |
| 1,73 | 0,43 | 1,86 | 7,00 | 7,00 | 16,33 | N/A | N/A | 3,17 | 2,38 | 1,38 | 1,90 | 1,00 | 6,81 | 2,00 |
| 1,42 | 0,73 | 1,97 | 3,74 | 0,00 | 20,19 | N/A | N/A | 2,03 | 1,87 | 1,13 | 1,87 | 0,00 | 7,32 | 1,15 |

**Table 2g:** Data extraction;

| **Author** | **tamponade** | **coronary malperfusion** | **hypovolaemic shock** | **bleeding** | **transitional care unit or rehabilitation** | **home** | **hospice** | **nursing home** | **other hospital** | **missing** |
| --- | --- | --- | --- | --- | --- | --- | --- | --- | --- | --- |
| 1 | N/A | N/A | N/A | N/A | N/A | N/A | N/A | N/A | N/A | N/A |
| 2 | N/A | N/A | N/A | N/A | N/A | N/A | N/A | N/A | N/A | N/A |
| 3 | N/A | N/A | N/A | N/A | 60,00 | 61,00 | 2,00 | 3,00 | 1,00 | 3,00 |
|  | N/A | N/A | N/A | N/A | 32,00 | 16,00 | 1,00 | 1,00 | 0,00 | 0,00 |
| 8 | 1,00 | 3,00 | 1,00 | 2,00 | N/A | N/A | N/A | N/A | N/A | N/A |
| 11 | N/A | N/A | N/A | 3,00 | N/A | N/A | N/A | N/A | N/A | N/A |
| 13 | N/A | N/A | N/A | N/A | N/A | 17,00 | N/A | N/A | 21,00 | N/A |
| 14 | N/A | 3,00 | N/A | N/A | N/A | N/A | N/A | N/A | N/A | N/A |
|  | N/A | 2,00 | N/A | N/A | N/A | N/A | N/A | N/A | N/A | N/A |
|  | N/A | 1,00 | N/A | N/A | N/A | N/A | N/A | N/A | N/A | N/A |
| 16 | N/A | 13,00 | N/A | N/A | N/A | N/A | N/A | N/A | N/A | N/A |
| 19 | N/A | N/A | N/A | N/A | N/A | 13,00 | N/A | N/A | N/A | N/A |
| 25 | N/A | 5,00 | N/A | 2,00 | N/A | N/A | N/A | N/A | N/A | N/A |
| 37 | N/A | N/A | N/A | N/A | N/A | N/A | N/A | N/A | 9,00 | N/A |
|  | 1,00 | 4,50 | 1,00 | 2,33 | N/A | N/A | N/A | N/A | N/A | N/A |
|  | 0,00 | 3,99 | 0,00 | 0,47 | N/A | N/A | N/A | N/A | N/A | N/A |

**Table 2h:** Data extraction; Assessment of quality of life

| **Author** | **physical funtioning** | **role limitations (physical)** | **energy level** | **emotional wellbeing** | **social functioning** | **pain** | **general health** |
| --- | --- | --- | --- | --- | --- | --- | --- |
| 3 | 55,00 | 100,00 | 52,50 | 78,00 | 90,00 | 70,00 | 53,50 |
|  | 70,00 | 100,00 | 47,50 | 90,00 | 90,00 | 90,00 | 55,00 |
| 11 | 60,00 | 43,25 | 53,15 | 61,64 | 51,88 | 27,80 | 58,05 |
| 30 | 48,09 | 32,90 | 48,20 | 57,90 | 56,90 | 55,80 | 51,70 |
| ∑ | 233,09 | 276,15 | 201,35 | 287,54 | 288,78 | 243,60 | 218,25 |
| Mean | 58,27 | 69,04 | 50,34 | 71,89 | 72,20 | 60,90 | 54,56 |
| SD | 7,98 | 31,18 | 2,51 | 12,90 | 17,89 | 22,65 | 2,33 |

**Table 3:** missings

|  | **age** | **pre shock** | **pre malperfusion** | **myocardial** | **limb** | **renal** | **cerebral** | **visceral** | **bmi** | **female** | **male** | **EuroSCORE II** | **DeBakey I** | **DeBakey II** | **imh** | **bav** | **marfan** | **acute type a** | **rescuscitation** | **rupture** | **tamponade** | **cad** | **pavk** | **av regurgitation more 2** | **av stenosis** | **ah** | **smoking** | **alcohol** | **diabetes** | **pre neurologic dysfunction** | **copd** | **renal failure** | **previous cardiac operation** | **pre AF** |
| --- | --- | --- | --- | --- | --- | --- | --- | --- | --- | --- | --- | --- | --- | --- | --- | --- | --- | --- | --- | --- | --- | --- | --- | --- | --- | --- | --- | --- | --- | --- | --- | --- | --- | --- |
| N | 10 | 23 | 35 | 45 | 48 | 52 | 41 | 50 | 47 | 40 | 39 | 57 | 50 | 48 | 57 | 52 | 44 | 56 | 43 | 53 | 38 | 42 | 49 | 47 | 59 | 33 | 46 | 56 | 41 | 50 | 40 | 38 | 46 | 52 |
| percentage | 0,17 | 0,38 | 0,58 | 0,75 | 0,8 | 0,87 | 0,68 | 0,83 | 0,78 | 0,67 | 0,65 | 0,95 | 0,83 | 0,8 | 0,95 | 0,87 | 0,73 | 0,93 | 0,72 | 0,88 | 0,63 | 0,7 | 0,82 | 0,78 | 0,98 | 0,55 | 0,77 | 0,93 | 0,68 | 0,83 | 0,67 | 0,63 | 0,77 | 0,87 |

| **congestive heart failure** | **previous myocardial infarction** | **previous stroke** | **pre creatinine** | **pre EF** | **isolated cerebral perfusion time** | **cerebral perfusion flow** | **ascending and hemiarch** | **elephant trunk** | **aortic root reconstruction** | **aortic root untouched** | **av conduit** | **av replacement** | **cabg** | **circulatory arrest** | **icu stay** | **ventilation time** | **reintubation** | **prolonged ventilation** | **chest tube drainage** | **reexploration for bleeding** | **length of stay** | **op duration** | **cpb** | **femoral cannulation** | **axillary cannulation** | **aorta cannulation** | **circulatory arrest time** | **crossclamp time** | **mean core temperature** | **total arch replacement** | **aortic root replacement** | **no post complications** | **infectious** |
| --- | --- | --- | --- | --- | --- | --- | --- | --- | --- | --- | --- | --- | --- | --- | --- | --- | --- | --- | --- | --- | --- | --- | --- | --- | --- | --- | --- | --- | --- | --- | --- | --- | --- |
| 56 | 48 | 47 | 55 | 52 | 49 | 59 | 29 | 58 | 37 | 55 | 41 | 33 | 30 | 40 | 36 | 50 | 54 | 43 | 52 | 37 | 35 | 34 | 32 | 47 | 50 | 52 | 9 | 23 | 24 | 18 | 18 | 58 | 37 |
| 0,93 | 0,8 | 0,78 | 0,92 | 0,87 | 0,82 | 0,98 | 0,48 | 0,97 | 0,62 | 0,92 | 0,68 | 0,55 | 0,5 | 0,67 | 0,6 | 0,83 | 0,9 | 0,72 | 0,87 | 0,62 | 0,58 | 0,57 | 0,53 | 0,78 | 0,83 | 0,87 | 0,15 | 0,38 | 0,4 | 0,3 | 0,3 | 0,97 | 0,62 |

| **sepsis** | **delirium** | **cerebral** | **bleeding** | **af** | **respiratory failure** | **renal failure requiring hemofiltration** | **tracheotomy** | **transient neurologic deficit** | **permanent neurologic deficit** | **postoperative stroke** | **gi bleeding** | **limb ischemia** | **myocardial infarction** | **los** | **readmission** | **30d mortality** | **in hospital death** | **late survival** | **multiorgan failure** | **respiratory failure** | **sepsis** | **ic hemorrhage** | **gi bleeding** | **los** | **aortic rupture** | **tamponade** | **coronary malperfusion** | **hypovolaemic shock** | **bleeding** | **transitional care unit or rehabilitation** | **home** | **hospice** | **nursing home** |
| --- | --- | --- | --- | --- | --- | --- | --- | --- | --- | --- | --- | --- | --- | --- | --- | --- | --- | --- | --- | --- | --- | --- | --- | --- | --- | --- | --- | --- | --- | --- | --- | --- | --- |
| 52 | 56 | 56 | 49 | 56 | 52 | 27 | 47 | 49 | 45 | 13 | 49 | 53 | 52 | 57 | 59 | 24 | 44 | 29 | 54 | 52 | 52 | 50 | 58 | 54 | 54 | 59 | 54 | 59 | 57 | 58 | 56 | 58 | 58 |
| 0,87 | 0,93 | 0,93 | 0,82 | 0,93 | 0,87 | 0,45 | 0,78 | 0,82 | 0,75 | 0,22 | 0,82 | 0,88 | 0,87 | 0,95 | 0,98 | 0,4 | 0,73 | 0,48 | 0,9 | 0,87 | 0,87 | 0,83 | 0,97 | 0,9 | 0,9 | 0,98 | 0,9 | 0,98 | 0,95 | 0,97 | 0,93 | 0,97 | 0,97 |

| **other hospital** | **missing** | **physical funtioning** | **role limitations (physical)** | **energy level** | **emotional wellbeing** | **social functioning** | **pain** | **general health** |
| --- | --- | --- | --- | --- | --- | --- | --- | --- |
| 56 | 58 | 56 | 56 | 56 | 56 | 56 | 56 | 56 |
| 0,93 | 0,97 | 0,93 | 0,93 | 0,93 | 0,93 | 0,93 | 0,93 | 0,93 |

**Table 4:** facts table; Data are expressed as n, mean ± standard deviation (SD), or percentage (%), as appropriate.

| variable | N/Mean (SD) | total cohort | percentage % |
| --- | --- | --- | --- |
| ***Baseline data*** |  |  |  |
| age | 77,92 |  |  |
| male | 714,00 | 1457,00 | 49,00 |
| female | 776,00 | 1447,00 | 53,60 |
| BMI (kg/m^2^) | 25± 3 |  |  |
| EuroSCORE II | 20± 16 |  |  |
| DeBakey I | 227,00 | 382,00 | 59,40 |
| DeBakey II | 202,00 | 474,00 | 42,60 |
| IMH | 36,00 | 93,00 | 38,70 |
| Marfans Syndrom | 8,00 | 1240,00 | 0,06 |
| acute type A dissection | 234,00 | 560,00 | 41,80 |
| CAD | 332,00 | 1664,00 | 20,00 |
| PAD | 159,00 | 674,00 | 23,60 |
| AH | 1721,00 | 2226,00 | 77,30 |
| DM | 140,00 | 1189,00 | 11,90 |
| COPD | 148,00 | 1228,00 | 12,00 |
| smoking history | 176,00 | 872,00 | 20,20 |
| alcohol history | 26,00 | 272,00 | 0,96 |
| renal failure | 133,00 | 1123,00 | 11,80 |
| congestive heart failure | 174,00 | 288,00 | 60,30 |
| AF | 96,00 | 614,00 | 15,60 |
| stroke | 75,00 | 652,00 | 11,50 |
| ***Preoperative data*** |  |  |  |
| preoperative shock | 552,00 | 2421,00 | 22,80 |
| preoperative tamponade | 534,00 | 1622,00 | 32,90 |
| preoperative CPR | 102,00 | 1767,00 | 0,60 |
| aortic rupture | 133,00 | 433,00 | 30,70 |
| average LVEF | 55,98 |  |  |
| malperfusion | 549,00 | 1781,00 | 30,80 |
| cerebral | 134,00 | 804,00 | 16,60 |
| renal | 55,00 | 408,00 | 13,50 |
| limb | 68,00 | 835,00 | 0,80 |
| visceral | 39,00 | 639,00 | 0,60 |
| ***Perioperative data*** |  |  |  |
| average cross-clamp time (min) | 104± 29 |  |  |
| average CPB (min) | 201± 58 |  |  |
| average circulatory arrest time (min) | 45± 42 |  |  |
| average temperature (°C) | 23± 3 |  |  |
| axillary cannulation | 234,00 | 777,00 | 30,00 |
| femoral cannulation | 457,00 | 1005,00 | 45,00 |
| central cannulation | 14,00 | 406,00 | 0,30 |
| total arch replacement | 483,00 | 3113,00 | 16,00 |
| hemiarch | 1048,00 | 2152,00 | 49,00 |
| root replacement | 328,00 | 3224,00 | 10,00 |
| root reconstruction | 903,00 | 1791,00 | 50,00 |
| root untouched | 15,00 | 356,00 | 0,40 |
| FET | 62,00 | 133,00 | 47,00 |
| Bentall | 129,00 | 1129,00 | 11,00 |
| AV replacement | 446,00 | 1955,00 | 23,00 |
| average ICU stay (d) | 11,00± 16,00 |  |  |
| average ventilation time (min) | 68,00± 34,00 |  |  |
| prolonged ventilation (>24h) | 39,00± 42,00 |  |  |
| chest tube drainage (ml) | 2,112.6 ± 1,808.0 |  |  |
| length of stay (d) | 24,00± 13,00 |  |  |
| ***Postoperative data*** |  |  |  |
| neurological transient complications | 40,00 | 596,00 | 0,70 |
| neurological permanent complications | 29,00 | 789,00 | 0,40 |
| delirium | 160,00 | 534,00 | 30,00 |
| stroke | 407,00 | 3506,00 | 12,00 |
| respiratory failure | 76,00 | 355,00 | 21,00 |
| renal failure | 421,00 | 1953,00 | 22,00 |
| septic shock | 38,00 | 378,00 | 10,00 |
| bleeding complications | 120,00 | 769,00 | 16,00 |
| new-onset AF | 49,00 | 193,00 | 25,00 |
| myocardial infarction | 15,00 | 525,00 | 0,30 |
| readmission to hospital | 7,00 | 68,00 | 10,00 |
| ***Postoperative mortality*** |  |  |  |
| LOS | 41,00 | 322,00 | 13,00 |
| bleeding complications | 7,00 | 202,00 | 0,30 |
| multiorgan failure | 19,00 | 372,00 | 0,50 |
| shock (cardiac, septic, hypovolaemic) | 12,00 | 462,00 | 0,30 |
| intracranial haemorrhage | 19,00 | 518,00 | 0,40 |
| aortic rupture | 12,00 | 294,00 | 0,40 |
| ***Quality of life*** |  |  |  |
| physical functioning | 58,00 ± 8,00 |  |  |
| role limitations | 69,00 ± 31,00 |  |  |
| vitality | 50,00 ± 3,00 |  |  |
| emotional well-being | 72,00 ± 13,00 |  |  |
| bodily pain level | 61,00 ± 23,00 |  |  |
| general health | 55,00 ± 2,00 |  |  |
